# Toward Fast‐Charging Polymer‐Electrolyte Based All‐Solid‐State Li–S Batteries: Insights into Limiting Factors and Perspectives

**DOI:** 10.1002/advs.75058

**Published:** 2026-04-09

**Authors:** S. Jayasubramaniyan, Mingxu Li, Hyeok‐jin Kwon, Sang Yong Nam, Younki Lee, Hyun Woo Kim

**Affiliations:** ^1^ Department of Chemical Engineering Gyeongsang National University Jinju Republic of Korea; ^2^ Research Institute for Green Energy Convergence Technology Gyeongsang National University Jinju Republic of Korea; ^3^ Department of Materials Engineering and Convergence Technology Gyeongsang National University Jinju Republic of Korea

**Keywords:** all‐solid‐state Li‐S battery, fast charge, high power, optimization strategies, polymer‐based solid electrolyte

## Abstract

The pursuit for high‐energy, fast‐charging all‐solid‐state lithium‐sulfur batteries (ASSLSBs) has intensified due to the increasing demand of next‐generation energy storage devices for electric vehicles. Polymer‐based solid electrolytes (PSEs) have distinct advantages, including mechanical flexibility, interfacial adaptability, and processability; however, their inherent limits in ionic conductivity, interfacial stability, and polysulfide shuttling impede fast charge‐discharge performance. This perspective scrutinizes the primary challenges influencing fast‐charging features of PSE‐based ASSLSBs, such as constrained lithium‐ion transport pathway, polysulfide shuttling, and elevated interfacial polarization. Also, the recent advancements in polymer molecular design, composite engineering, and interfacial modification are outlined, highlighting approaches to attain high ionic conductivity, increased Li‐ion transference number, and stable electrode–electrolyte interfaces are addressed. Further, research directions for adaptive, high‐rate ASSLSBs are explored, including design strategies for increasing the ionic conductivity, mitigating polysulfide shuttling and designing a stable interface. Moreover, a comprehensive design framework that incorporates ion‐transport optimization, chemical selectivity, and interface engineering is proposed to facilitate stable and dendrite‐free fast‐charging ASSLSBs. We believe this perspective offers a comprehensive overview of the progression of PSEs for practical, high‐power Li‐S batteries, connecting laboratory advancements with practical applications.

## Graphical abstract

The schematic illustrates the important design strategies for polymer solid electrolyte‐based all‐solid‐state lithium‐sulfur batteries (ASSLSBs), including improved ionic conductivity, reduced polysulfide shuttling, stabilized electrode‐electrolyte interfaces, and lithium dendrite mitigation for developing high power and fast charging ASSLSBs.

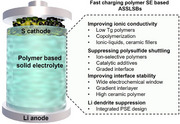

## 1 Introduction

**FIGURE 1:**
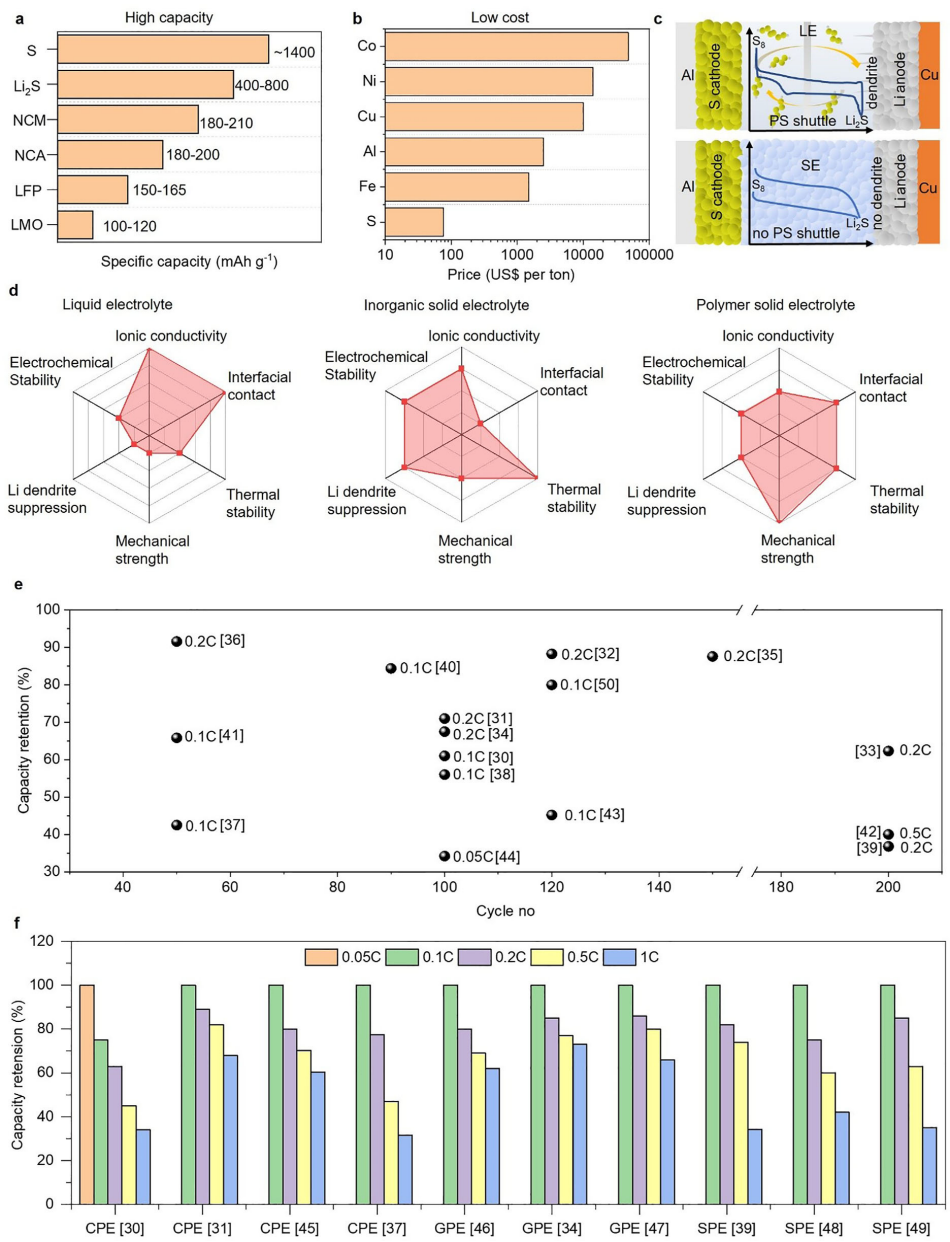
(a–c) advantages of sulfur cathode in ASSBs. (d) advantages of PSEs over the organic liquid and inorganic solid electrolytes. (e) cycle life and (f) rate retention trend comparison of polymer‐based SE in ASSLSBs. Note that data presented in (e,f) are compiled from research studies conducted under different testing conditions (e.g., sulfur loading, different PSEs, C‐rates, and operating temperature; see supporting information Table S1). Thus, these comparisons are intended to indicate the overall performance tendencies in cycling durability and rate capability of PSE‐based ASSLSBs, rather than to offer a direct quantitative comparison.

The rapid adoption of electric vehicles and portable electronic devices has substantially increased the need for advanced energy storage technologies. Rechargeable lithium batteries, known for their extended cycle life, lightweight design, and absence of memory effect, proficiently fulfill these requirements. As a result, there is an intensive attempt to improve their specific capacity to satisfy the increasing energy density demands across various applications [1]. Lithium‐ion batteries (LIBs) have gained prominence in the last two decades, especially for portable devices, due to their significantly high energy density compared with other rechargeable systems such as lead‐acid and Ni‐Cd. However, the currently available LIB technology relies on insertion‐compound anode and cathode materials, which limit their charge‐storage capacity and energy density (300 Wh kg^−1^). This performance is inadequate to satisfy the rapidly growing market demand for high‐power energy storage systems [2, 3, 4, 5]. To attain high power and energy density, it is essential to increase the charge‐storage capacity of both anode and cathode materials, elevate the cell voltage, or implement a combination of these approaches is necessary. To meet the necessity for increased energy density in various power tools, a lithium‐sulfur (Li‐S) battery, with a potential theoretical energy density of 2600 Wh kg^−1^, is considered the most suitable option [6, 7]. Sulfur provides a theoretical capacity of 1672 mA h g^−1^, far surpassing that of transition‐metal oxide cathodes. The elevated capacity derives from the conversion reaction of sulfur into lithium sulfide (Li_2_S), which involves the reversible incorporation of two electrons per sulfur atom, in contrast to one or fewer electrons per transition‐metal ion in insertion‐oxide cathodes (Figure 1a) [8]. Further, sulfur is advantageous because of its abundant resources, cost‐effectiveness, and safety for humans (Figure 1b). As a result, Li‐S batteries possess significant potential for advancement and use in next‐generation energy storage systems.

Despite their immense promise, Li‐S batteries experience numerous significant obstacles that have impeded their commercialization. One of the most important problems is that sulfur has a very low electronic conductivity (about 5 × 10^−30^ S cm^−1^), and its discharge products, Li_2_S and Li_2_S_2_, are insulators [9, 10]. The internal resistance of the cell is raised by these factors, which lower overall performance. Another significant challenge is the shuttle effect, wherein lithium polysulfides (LiPSs) dissolve in the liquid electrolyte (LE) during cycling and migrate through the separator to the lithium anode. This leads to the permanent depletion of active sulfur, diminished sulfur utilization efficiency, and the gradual capacity loss upon continuous cycling [11, 12]. Also, the liquid electrolyte‐based Li‐S systems, typically utilizing ether‐based electrolytes, encounter safety hazards, including leakage, flammability, inadequate thermal and chemical stability, and the risk of explosions [13]. In addition, the transformation of sulfur to Li_2_S during discharge results in 80% volumetric expansion of the electrode followed by a contraction during the charging process. This expansion and contraction deteriorate the contact among the active material, conductive binder, and electrolyte, which causes electrical contact loss and leads to poor capacity retention [14, 15]. The challenges of poor electronic conductivity intermediates, structural instability, the shuttle effect, and electrolyte‐related safety hazards collectively contribute to capacity degradation and diminished cycle stability.

Several approaches have been studied to address the inherent constraints of liquid electrolytes (LEs), such as safety concerns, polysulfide shuttling, and poor interfacial stability. Solid electrolytes (SEs) have emerged as a feasible alternative to LE owing to their increased safety, high thermal stability, and ability to enable high‐energy‐density Li‐S batteries [16, 17, 18]. In addition, the utilization of sulfur in solid‐state batteries has additional advantages over the conventional Li‐S battery. In conventional Li‐S batteries, the electrochemical reduction of sulfur occurs via a multistep solid‐liquid‐solid transformation, following the overall reaction S + 2Li + 2e^−^ → Li_2_S. During discharge, sulfur initially undergoes reduction to form long‐chain soluble lithium polysulfides (Li_2_S_x_, where 6 ≤ x < 8; LiPSs), which dissolve in the electrolyte and are subsequently further reduced to short‐chain species before precipitating as solid Li_2_S. This intricate sequence results in the two‐plateau discharge profile. Conversely, Li‐S batteries utilizing SEs operate based on a fundamentally different mechanism. Because LiPSs are unable to dissolve or migrate within the solid matrix, sulfur experiences a direct, single‐phase solid‐state transformation to Li_2_S without the formation of mobile LiPS intermediates and exhibits a single plateau discharge profile (Figure 1c) [19, 20, 21, 22]. SEs to operate efficiently as electrolytes, they must meet certain essential criteria: (1) high ionic conductivity at ambient temperature (10^−4^–10^−3^ S cm^−1^) for enabling fast charge‐discharge performance; (2) high lithium‐ion transference number (t_Li_^+^) for mitigating bulk ion concentration gradients at interfaces, reducing the risk of dendrite formation, and extending battery durability; (3) superior mechanical strength, particularly at a high shear modulus (>6 GPa) to efficiently prevent lithium dendrite penetration; (4) high chemical and electrochemical compatibility for ensuring minimal interfacial resistance; and (5) high thermal stability for good safety [23]. Materials suitable for SEs encompass solid polymer electrolytes (SPEs), gel polymer electrolytes (GPEs) composite polymer electrolytes (CPEs), and inorganic solid electrolytes (ISEs). Polymer‐based solid electrolytes (PSEs) have significant advantages compared to LEs and inorganic‐based electrolytes for Li‐S batteries (Figure 1d). They exhibit reduced flammability and increased mechanical robustness over LE, thereby substantially improving battery safety. Also, PSEs demonstrate enhanced flexibility over inorganic SE materials, which enhances electrode contact and accommodates volume fluctuations during cycling [24]. Remarkably, their composition, microstructure, and intrinsic ion transport properties can be tuned through methods like chemical structural modification, side‐chain inclusion, or polymer blending [25]. In addition, polymers can be integrated with fillers or ionic liquids to produce composite SE material, facilitating the Li‐ion transport and physicochemical properties of PSE [26, 27].

Moreover, the advancement of high‐power, fast‐charging all‐solid‐state Li‐S batteries (ASSLSBs) represents a significant milestone in the progression of next‐generation energy storage devices for electric vehicles (EVs) aimed at integrating ultrahigh energy density, fast energy discharge, and improved safety. To ensure EVs are competitive with traditional refueling time, the U.S. Advanced Battery Consortium set a goal for 2023 of attaining an 80% state of charge (SOC) within 15 min of charging time. Achieving this benchmark is essential for alleviating range anxiety and improving user convenience. However, such rapid recharge times require exceedingly high charging power, approximately 300 kW for large battery packs (e.g., 90 kWh) and around 80 kW even for smaller packs (e.g., 24 kWh) [28, 29]. These requirements pertain to charging rates of 4C or greater, emphasizing that the fast‐charging capability of the battery is an essential prerequisite for widespread electric vehicle adoption. Li‐S batteries have a high theoretical energy density; however, the actualization of this potential is hindered by inherent material and interfacial issues, such as the insulating properties of sulfur and Li_2_S, sluggish redox kinetics, poor ionic conductivity of PSEs, electrochemical instability at the interface and the migration of soluble polysulfide intermediates, which together result in inadequate cycle stability, rate capability, and fast capacity degradation even at the low C‐rates (Figure 1e, f, and Table S1) [30, 31, 32, 33, 34, 35, 36, 37, 38, 39, 40, 41, 42, 43, 44, 45, 46, 47, 48, 49, 50]. These issues become most pronounced during high‐rate or rapid charging conditions, where restricted ionic mobility and unstable electrode‐electrolyte interfaces lead to substantial voltage polarization and mechanical deterioration. Therefore, a comprehensive understanding of the essential parameters influencing ion transport, interfacial kinetics, and electrochemical stability in PSEs is critically required to mitigate the limitations and for developing fast‐charging PSE‐based ASSLSBs.

This perspective seeks to address the limitation in the development of fast‐charging PSEs‐based ASSLSBs by critically examining the significant key limiting factors, including sluggish Li‐ion transport, persistent polysulfide shuttling, and interface‐related impediments of PSEs. By systematically integrating recent developments in PSE design to address the limitations, this perspective aims to provide a comprehensive framework of design strategies and emerging research directions that could enable the next generation of high‐power, durable, and fast‐charging PSE‐based ASSLSBs. We anticipate that this perspective offers significant insights into the underlying influencing factors, dominant failure mechanisms, and advanced optimization strategies essential for attaining high power density and high current operation in PSE‐based next‐generation ASSLSBs, thus facilitating the connection between fundamental research and practical applications.

## 2 Challenging Factors for Achieving Fast‐Charge in Polymer‐Based SE

**FIGURE 2:**
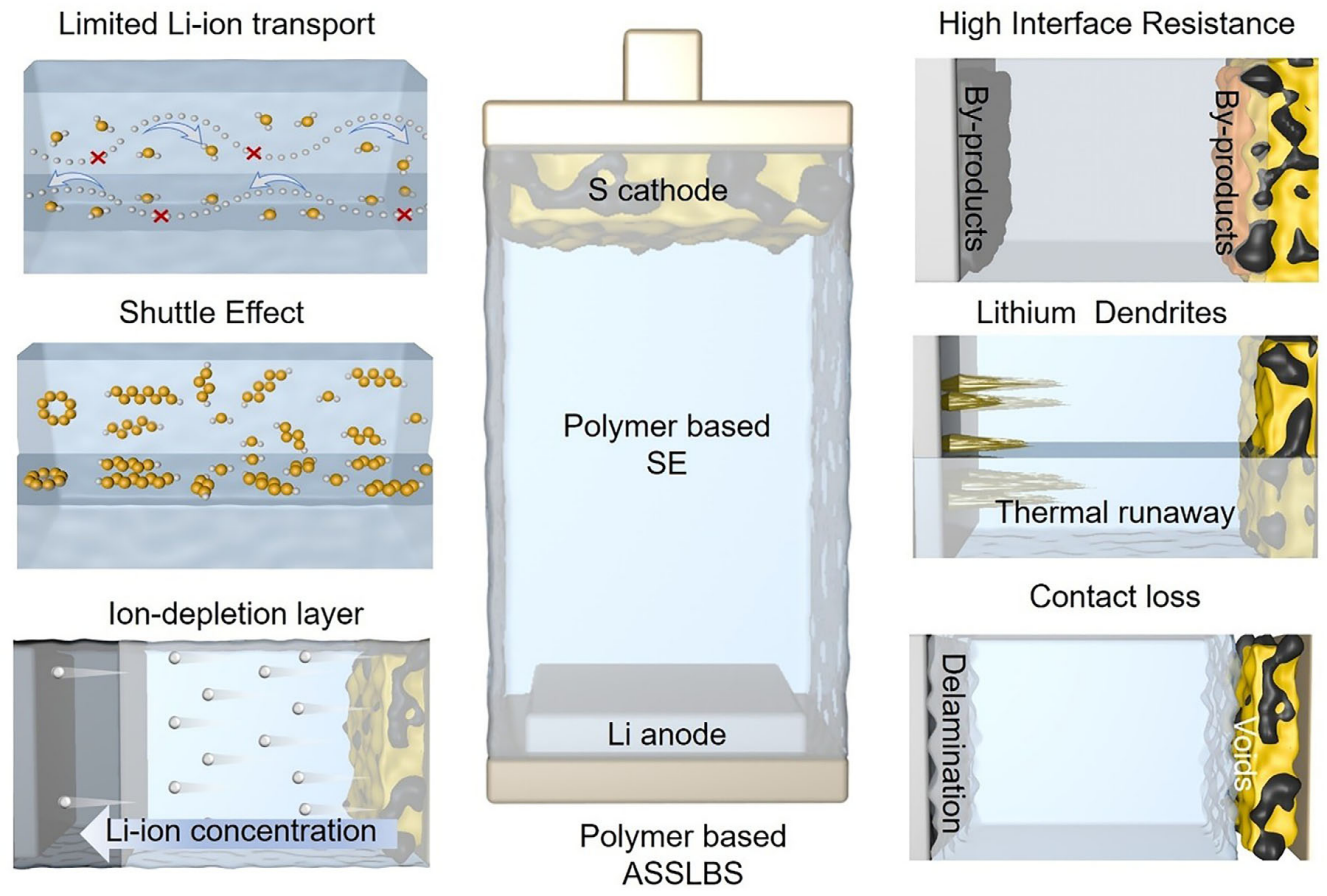
Limiting factors to achieve fast charging of polymer‐based SE in ASSLSBs.

PSEs provide numerous advantages, including flexibility, ease of processing, and increased safety compared to ether‐based liquid electrolytes; yet, achieving fast‐charging performance in ASSLSBs remains a significant challenge. The key challenges that limit PSEs utilization under fast‐charging conditions are limited Li‐ion transport, polysulfide shuttling, unstable electrode‐electrolyte interfaces, mechanical deformation, and dendrite growth (Figure 2). These issues lead to uneven Li‐ion flux, formation of ion‐depletion layer, considerable interfacial polarization, and rapid capacity decline, making it challenging for ASSLSBs to provide stable performance at high current density [24]. Therefore, a comprehensive understanding of these challenges and failures are essential for designing effective PSEs, which can provide a reasonable performance at elevated current densities. This section provides a detailed discussion of the main challenging factors that limit the fast‐charging and high‐power performance of PSE‐based ASSLSBs.

### 2.1 Ionic Conductivity Limitation of PSEs

The ionic conductivity of solid electrolytes is an important parameter in the development of fast‐charging batteries, as low conductivity causes high internal resistance, increased polarization, and poor ion transport at high current densities [51]. Generally, PSEs demonstrate inherently low Li‐ion conductivity (10^−6^ to 10^−5^ S cm^−1^ at ambient temperature) as lithium transport is closely linked to polymer segmental motion. In common host polymers like polyethylene oxide (PEO), polyvinylidene fluoride (PVDF), or polyacrylonitrile (PAN), a substantial portion of the matrix assumes semi‐crystalline or glassy forms at room temperature, where polymer chain mobility is markedly inhibited. Hence, the Li‐ion conduction predominantly occurs through coordination–dissociation events involving ether or polar functional groups on the polymer backbone, where constrained segmental dynamics significantly reduce ion hopping frequencies and increase the activation energy for long‐range ion migration [52, 53]. Moreover, the robust Lewis acid–base interactions between Li‐ion and coordinating sites tend to localize ions and facilitate the formation of ion pairs or multi‐ion aggregates, hence reducing the number of dissociated species available for charge transport [54]. These intrinsic features collectively form a critical bottleneck in PSEs, diminishing overall electrochemical performance of the cell during high C‐rate operations.

In addition to the impact of bulk ionic conductivity, the Li‐ion transference number (t_Li_^+^) of PSEs is an essential factor that determines the fast‐charging performance of ASSLSBs. In typical PSEs, including PEO, Li‐ion transference number is relatively low, typically ranging from ∼0.2 to 0.4, indicating that a significant portion of the ionic current is carried by anions instead of Li‐ions. This arises due to strong coordination of Li‐ion with polar groups in the polymer chains (e.g., ether oxygens), which limits its mobility, whereas the anions exhibit relatively more mobility [55, 56]. Consequently, with fast charging, substantial concentration polarization occurs within the electrolyte and Li‐ions ions are depleted near the electrode while anions aggregate in other regions [57] This results in increased overpotential, decreased effective Li‐ion transport, and limited rate capability. The synergistic impact of low ionic conductivity and a low Li‐ion transference number of PSEs results in poor Li‐ion flux under higher charging currents, limiting PSEs from facilitating fast‐charge conditions without substantial polarization or potential risks, including lithium plating.

### 2.2 Polysulfide Shuttling in PSEs

The polysulfide shuttle effect is significantly reduced in PSEs relative to ether‐based liquid electrolyte systems, as the diffusion coefficients of LiPS in PEO‐based PSE (10^−^^8^–10^−^^9^ cm^2^ s^−1^) are generally 2–3 orders of magnitude lower than those in conventional ether‐based LEs (∼10^−^^6^–10^−^^7^ cm^2^ s^−1^) [11, 58]. However, it continues to pose a significant challenge, particularly under fast charging conditions in ASSLSBs. The partial solvation and migration of LiPSs are facilitated by the significant chain segment mobility and polar functional groups present in many polymers, which reducing the energetic barrier for crossover and allows for LiPSs diffusion into the polymer electrolyte matrix [59]. This issue becomes more severe when the operating temperature and the C‐rate increase, as the transfer of LiPSs is facilitated by pronounced concentration gradients and increased segmental motion. The subsequent migration leads to a significant reduction in capacity by reducing the active sulfur utilization [60]. Additionally, parasitic interactions with the lithium‐metal anode produce insulating layers (Li_2_S/Li_2_S_2_) that destabilize the interface and increases the interface resistance. Even composite polymer electrolytes are unable to completely mitigate this phenomenon due to the elevated solubility of LiPSs in polymer matrices, which leads to self‐discharge during fast‐charging conditions [61] The rate capability and cycle longevity of PSE‐based ASSLSBs are ultimately compromised by continuous shuttling, which induces side reactions, creates concentration gradients, elevates interfacial impedance, and expedites capacity degradation during fast charging.

### 2.3 Interface Stability Issues of PSEs

Despite significant developments, PSEs continue to face enormous interfacial challenges, leading to a substantial increase in the charge‐transfer resistance at the interface, rapid capacity deterioration, and poor long‐term cycling stability at high C‐rates. At the PSE/cathode interface, sulfur conversion processes at the cathode interface result in soluble polysulfides, which are stabilized by polar groups in the polymer matrix and easily diffuse into the polymer electrolyte [23]. By actively participating in redox processes, these species assault the polymer chemically and produce an unstable, heterogeneous interphase. At the same time, interfacial resistance increases, and Li‐ion transport is blocked by the development of insulating Li_2_S/Li_2_S_2_ deposits [24]. In addition, sulfur redox causes large volume changes that further obstruct mechanical contact between the polymer and cathode, resulting in voids, delamination of the electrode from the current collector, and transport bottlenecks that deteriorate performance and increase polarization [25]. At the PSE/lithium anode interface, uneven lithium deposition under high C‐rates leads to an increase in the localized current regions, thus promoting dendrite formation and interfacial instability. Concurrently, uneven lithium plating and volumetric changes at the electrode/electrolyte interface decrease adhesion and resulting stress accumulation led to interfacial void formation and delamination, resulting in increased charge‐transfer resistance, poor contact, and expedited capacity degradation. Consequently, preserving interfacial mechanical integrity is equally crucial as enhancing ionic conductivity for facilitating fast‐charging ASSLSBs. In addition, the chemical incompatibility issues between PSE and Li metal trigger side reactions that continuously elevate the charge transfer resistance, leading to substantial polarization, thus limiting the fast charge features of the PSE‐ based Li‐S batteries [26, 27].

### 2.4 Li Dendrite Growth

In PSE‐based ASSLBS, the formation of lithium dendrites and low critical current density (CCD) collectively establish a significant constraint on the feasible fast‐charging performance by closely linking electrochemical, transport, and mechanical limitations [62]. Dendrite formation occurs when the applied charging current surpasses the capability of the polymer to maintain a uniform spatial flux of lithium ions and concurrently resist localized mechanical intrusion at the Li/polymer interface [63]. Under these conditions, Li‐ion depletion, pronounced concentration gradients, and significant intensification of interfacial electric fields occur, resulting in preferential lithium nucleation at the areas of increased local conductivity. In addition, practical fast charging (4C charging rate) typically requires CCD values above 2 mA cm^−^^2^ to ensure uniform lithium plating and stripping without the risk of dendrite formation [64]. However, a majority of PSEs exhibit relatively low CCDs, often ranging from 0.1 to 0.5 mA cm^−^^2^, consequently limiting their capacity for supporting high‐rate charging in practical cell [44]. This discrepancy between the necessary and sustainable current density leads to expedited voltage polarization, facilitates fast dendrite growth within the flexible polymer matrix, and ultimately induces internal short‐circuiting [65]. This issue is further aggravated by polysulfide residues and sulfur‐derived interphase species, which decrease the Li‐ion transference number and chemically compromise the interfacial region [66]. Collectively, these effects hinder PSE‐based Li‐S batteries from attaining reliable, safe fast‐charging capabilities unless novel polymer architectures are engineered to incorporate high mechanical stiffness, increased Li‐ion transference number, reinforced or hybrid frameworks, and decoupled ion‐hopping or ion‐channel‐based transport mechanisms to maintain stable, dendrite‐free Li plating at elevated current densities.

In addition, PSEs pose considerable safety challenges during high current operation owing to their limited thermal conductivity, which leads to the localized heat generation at the electro‐electrolyte interface and promotes the uneven Li deposition and dendrite growth, resulting in an internal short circuit. Concurrently, the low thermal conductivity of PSEs limits the heat dissipation under fast charging conditions, triggering the formation of and propagation of isolated hotspots [67, 68]. Thus, the combination of dendrite‐induced short circuits, interfacial resistance heating, and thermally induced electrolyte decomposition increases the cell temperature, resulting in thermal runaway. Consequently, ensuring thermal stability and inhibiting dendrite development is a substantial challenge for PSEs operating under fast charging conditions.

## 3 Current Strategies for Fast‐Charging Polymer Based ASSLSBs

### 3.1 Improving Ionic Conductivity of Polymer‐Based SE

PSEs are required to exhibit high ionic conductivity, ideally between 10^−2^ and 10^−3^ S cm^−1^ at ambient temperature, to reduce internal resistance and facilitate efficient Li‐ion transfer. In PSEs, Li‐ion conduction occurs by a coordination‐hopping mechanism that is closely linked to the polymer segmental motion. Li‐ ions are solvated by the polar functional groups of the polymer and transport primarily happening in the amorphous phase. The inherent tendency of PSEs to crystallize limits ionic transport. To address this limitation, various modification strategies have been developed, including the use of the incorporation of inorganic fillers, different lithium salt additives, cross‐linking techniques, and the creation of organic–inorganic hybrid systems, all aimed at reducing crystallinity and enhancing ionic conductivity.

#### 3.1.1 Composite Polymer Electrolytes

**FIGURE 3:**
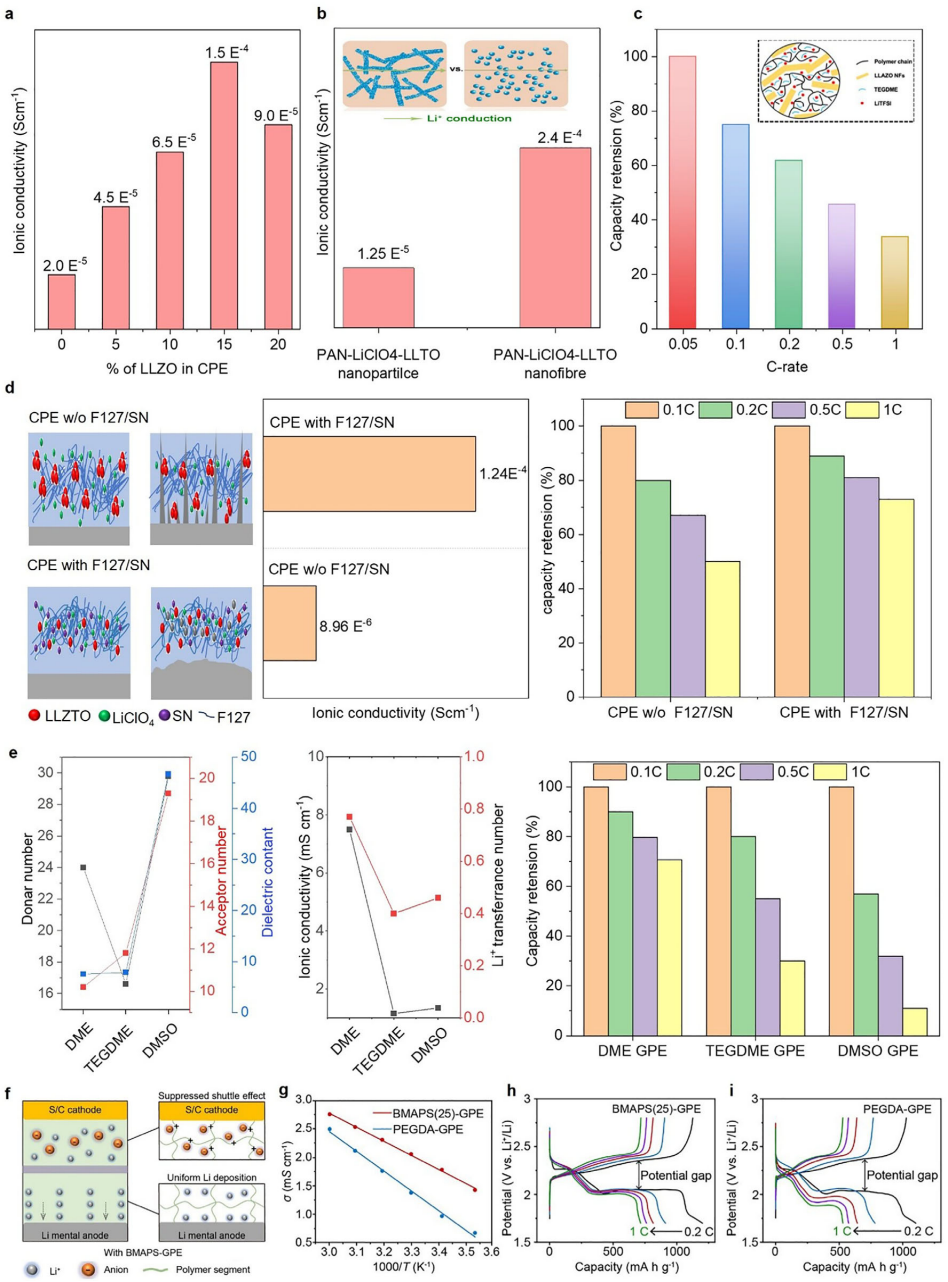
Ionic conductivity improvement strategies to achieve high‐rate capability. (a) Influence of ceramic filler composition on the ionic conductivity of CPE at 60°C. Figure adapted with permission from ref. [70] Copyright 2017, American Chemical Society. (b) Influence of ceramic filler morphology on the ionic conductivity of CPE at room temperature. Figure reproduced with permission from ref. [72] Copyright 2015, American Chemical Society. (c) Rate retention trend of CPE containing LLAZO nanofibers in ASSLASBs tested at room temperature. Figure reproduced with permission from ref. [30] Copyright 2024, American Chemical Society. (d) Influence of dispersant and plasticizer on the ionic conductivity and rate retention behavior of PEO/LLZTO‐based CPE in ASSLSBs tested at room temperature. Figure adapted with permission from ref. [31] Copyright 2025, American Chemical Society. (e) Influence of solvent properties such as DN, AN and dielectric constant on the ionic conductivity and rate retention behavior of GPE in ASSLSBs tested at 25°C. Figure adapted with permission from ref. [32] Copyright 2025, American Chemical Society. (f–i) Ionic conductivity and rate retention behavior of the GPE containing zwitterionic polymer tested at room temperature. Figure reproduced with permission from ref. [82] Copyright 2025, Wiley‐VCH.

Composite polymer electrolytes that incorporate inorganic particles into polymer matrices represent a highly promising approach, since they combine the advantages of SPEs and ICEs. The polymer component optimizes interfacial compatibility with electrodes and provides mechanical and thermal resilience, while the ceramic phase increases ionic conductivity. However, when crystalline solids and polymers are simply combined, ceramic particles often segregate inside the polymer matrix, leading to Li‐ion transport mostly via the polymer/ceramic interface. This limitation hinders the overall efficiency of Li‐ion movement within the CPE, which ultimately reduces the fast‐charging kinetics of the ASSLSBs [22, 69]. Numerous research works have been carried out to increase the ionic conductivity of the CPE. Increasing the ceramic content in CPEs substantially improves interfacial compatibility and establishes continuous conductive pathways, thereby enhancing lithium‐ion transport, which leads to improved rate retention [26]. For instance, Tao et al. [70] reported that the incorporation of a higher percentage of Li_7_La_3_Zr_2_O_12_ (LLZO) ceramic fillers into the PEO polymer enabled the establishment of uninterrupted Li‐ion conduction channels inside the polymer matrix due to the intrinsic high ionic conductivity of LLZO and its strong Lewis acidity, which enhances lithium salt dissociation and ion mobility. The uniformly distributed LLZO nanoparticles suppressed the crystallinity of the PVDF phase, creating a more amorphous phase that enhanced the segmental motion of the polymer chains and accelerated Li‐ion transport. As a result, the ionic conductivity markedly increased to 1.5 × 10^−4^ S cm^−1^ for the 15wt% LLZO/PEO CPE at 60°C (Figure 3a). Nonetheless, the ionic conductivity decreases gradually as the LLZO content is elevated to 20 wt%. At increased loadings, LLZO particles generally tend to aggregate and promote micro‐phase separation or void formation, resulting in additional interfaces that hinder continuous Li‐ion transport.

Compared to the simple composite method, rational structural design of ceramic filler in CPEs has emerged as a viable strategy for improving the ionic conductivity. Nanosized or well‐dispersed ceramic fillers with a high surface area provide extensive interfacial contact area between the ceramic and polymer, promoting the formation of continuous Li‐ion pathways and improving ion dissociation from lithium salts. Moreover, one‐dimensional (nanowires) and two‐dimensional (nanosheets) ceramic structures provide directed and percolating channels for rapid Li‐ion migration, enhancing connection throughout the electrolyte [69, 71]. Therefore, optimizing the dimension, morphology, and distribution of ceramic fillers is crucial for enhancing ionic conductivity, reducing activation energy for Li‐ion transport, and assuring consistent ion flux inside composite polymer electrolytes. For instance, Liu et al. [72] reported that the ionic conductivity of CPE is highly influenced by the morphology of the LLTO ceramic filler (Figure 3b). Specifically, integrating high‐aspect ratio LLTO nanowires into a PAN‐LiClO_4_ matrix establishes continuous, percolated Li‐ion transport pathways, resulting in an impressive room‐temperature conductivity of 2.4 × 10^−4^ S cm^−1^, which is nearly three orders of magnitude higher than that of the polymer alone. In contrast, LLTO nanoparticles containing CPE offer only modest enhancements in the Li‐ion conductivity compared to the pristine PAN‐LiClO_4_ matrix due to their isolated, discontinuous structure, which results in increased charge transfer resistance and restricted Li‐ion pathway connectivity.

In addition, Cheng et al. [30] demonstrated that the addition of electrospun Li_6.28_La_3_Al_0.24_Zr_2_O_12_ (LLAZO) nanofibers into a PEG‐based CPE significantly improved Li‐ion conductivity and rate performance. The one‐dimensional LLAZO nanofibers established a continuous inorganic‐organic framework that facilitated fast Li‐ion transport channels, yielding a high ionic conductivity of 5.71 × 10^−4^ S cm^−1^ at room temperature, a low activation energy of 0.268 eV, and an increased Li‐ion transference number of 0.70. Also, the strong Lewis acid–base interactions between LLAZO and LiTFSI facilitated ion dissociation and hindered anion migration. As a result, ASSLSB demonstrated exceptional rate capability at elevated current densities (Figure 3c). Further, Wang et al. [31] reported that the incorporation of both dispersant (Pluronic F127) and plasticizer (succinonitrile, SN) synergistically improved the ionic conductivity and rate retention of the PEO/LLZTO‐based CPE. The dispersant F127 successfully inhibited the agglomeration of LLZTO ceramic particles, resulting in a more homogeneous distribution and a continuous ion‐transport network inside the polymer matrix. Simultaneously, the plasticizer SN reduced the crystallinity of PEO, thus increasing the amorphous part of the electrolyte where Li‐ion migration primarily occurs. SN also promoted the dissociation of lithium salts by strongly coordinating with Li‐ion through its polar functional groups, thereby contributing to greater Li‐ion mobility. As a result, the co‐assisted CPE attained a much superior ionic conductivity of 1.24 × 10^−4^ S cm^−1^ at ambient temperature, in contrast to 8.98 × 10^−6^ S cm^−1^ for the unmodified CPE. In addition, the modified CPE showed high Li‐ion transference number of 0.541 which is significantly higher than the unmodified CPE (0.493). The enhanced ion transport eased accelerated charge transfer and decreased polarization during high C‐rate operation, yielding a high‐rate performance (Figure 3d). The synergistic roles of the dispersant and plasticizer, particle dispersion, and crystallinity suppression significantly enhanced the ionic conductivity and electrochemical durability of the Li‐S batteries.

Moreover, the composite polymer‐based quasi‐solid‐state electrolyte (QSSE) using ionic liquids (ILs) is an advanced electrolyte system for achieving high ionic conductivity and Li‐transference number in CPEs [73, 74, 75]. For instance, Pang et al. [76] demonstrated that incorporating a small amount of LE into a PEO/LLZTO‐based quasi‐solid‐state electrolyte significantly improves ionic conductivity (0.8 × 10^−3^ S cm^−1^ at ambient condition) and high Li‐ion transference number of 0.62 by plasticizing the polymer matrix, enhancing interfacial wettability, and establishing continuous Li‐ion transport pathways. The enhanced ionic conductivity, elevated Li‐ion transference number and diminished interfacial resistance directly contribute to improved electrochemical kinetics. At high current densities, Li‐ion transport is no longer to be the primary limiting factor, thereby enabling more comprehensive sulfur redox reactions. As a result, QSSE‐based Li–S batteries exhibit improved rate retention and faster reaction reversibility.

#### 3.1.2 Gel Polymer Electrolyte

Gel polymer electrolytes (GPEs) have a solid polymer matrix and a liquid electrolyte that is made up of a solvent and lithium salt. The liquid phase enhances lithium‐ion conductivity, facilitates ion transport, and reduces polysulfide solubility, thereby accelerating the kinetics of the sulfur redox reaction (SRR) and enabling stable cycling [77]. At the same time, the polymer framework serves as a physical barrier that reduces the shuttle effect and improves rate retention [78]. However, its ionic conductivity is hindered by the properties of the solvents, such as dielectric constant (ε), donor number (DN), and acceptor number (AN). For instance, Gomes et al. [32] conducted a comprehensive investigation of the solvent‐dependent ion transport characteristics in polycaprolactone (PCL)‐based GPEs for Li–S batteries, comparing dimethyl sulfoxide (DMSO), 1,2‐dimethoxyethane (DME), and tetraethylene glycol dimethyl ether (TEGDME) systems (Figure 3e). The results showed that the DME‐based GPE had the highest ionic conductivity (7.49 × 10^−4^ S cm^−1^ at 25°C) and Li‐ion transference number (0.727), as well as the lowest activation energy (8.7 kJ mol^−1^). These improvements were due to the low crystallinity of GPE (2.31%), which was caused by the great compatibility between PCL and DME that facilities the segmental polymer mobility and efficient Li‐ion hopping. The rate performance of several GPEs showed that the DME‐based system had the best capacity recovery (95.1%) when cycled from 0.1 to 1 C. This was better than the TEGDME‐ (71.6%) and DMSO‐based (54.6%) systems owing to the modest donor number and low viscosity of DME, which allow for effective Li‐ion solvation, increased ionic mobility. The increased viscosity of TEGDME and stronger Li‐ion binding slow down ion transport at high rates. On the other hand, the large donor number of DMSO encourages parasitic reactions and radical generation that make the SEI less stable and lower the rate performance.

The utilization of zwitterionic gel polymer electrolytes (ZGPEs) significantly improves the performance of ASSLSBs even under low‐temperature conditions. The ZGPEs contain both cationic and anionic groups, which promote the dissociation of electrolyte salts through effective electrostatic interactions, thereby increasing the concentration of mobile Li‐ions and maintaining relatively high ionic conductivity, even at low temperatures [79]. Further, the strong electrostatic interaction of the positively charged functional groups in ZGPEs with electrolyte anions hinders the anion migration, thus improving the Li‐ion transference number and mitigating concentration polarization [80]. Moreover, the functional groups of ZGPEs electrostatically interact with LiPS species, inhibiting their diffusion and decreasing the polysulfide shuttle effect, hence enhancing cycling stability and rate retention [81] Consequently, ZGPEs provide more stable and efficient operation of Li–S batteries at low temperatures. For instance, Li et al. [82] designed a ZGPE that demonstrates remarkably high ionic conductivity and excellent rate retention in Li‐S batteries (Figure 3f–i). The optimized 3‐[bis[2‐(methacryloyloxy)ethyl](methyl)ammonio]propane‐1‐sulfonate BMAPS(25)‐GPE exhibits an excellent ionic conductivity of 1.43 × 10^−3^ S cm^−1^ at a significantly low temperature of 10°C markedly surpassing the poly(ethylene glycol) diacrylate (PEGDA)‐based GPE (Figure 3g). This improvement is attributed to enhanced salt dissociation and a reduced Li‐ion migration barrier (Ea = 0.14 eV). The BMAPS(25)‐GPE also attains a high Li‐ion transference number (t_Li_^+^ = 0.72), effectively mitigating concentration polarization and enhancing interfacial lithium deposition. When applied in Li–S cells, the BMAPS(25)‐GPE exhibits exceptional rate capability, surpassing the performance of the PEGDA‐GPE counterpart (Figure 3h,i). The robust rate retention is ascribed to high ionic conductivity of GPE, enhanced Li‐ion diffusion coefficient, and reduced charge‐transfer resistance (R_CT_ = 52 Ω), which collectively enhance sulfur cathode reaction kinetics and diminish polysulfide shuttle effects. Overall, a highly solvating Li‐ion environment, produced by solvents with elevated donor numbers or dielectric constants, may accelerate sulfur redox kinetics at the cathode while concurrently compromising the stability of the Li metal anode. Consequently, attaining an optimal balance in solvent properties is crucial and should be a main focus in the systematic design of next‐generation Li‐S batteries.

#### 3.1.3 Solid Polymer Electrolyte

Solid polymer electrolytes (SPEs), which are fabricated by dissolving lithium salts into a polymer matrix, represent a prospective alternative to LE owing to their enhanced safety. However, existing SPEs demonstrate comparatively low ionic conductivity at ambient temperature (10^−6^–10^−4^ S cm^−1^) in comparison to liquid electrolytes (10^−3^–10^−2^ S cm^−1^), which constrains rate performance, particularly under fast charging conditions. Crosslinking has proven to be an efficient method for addressing the inherent trade‐off between ionic conductivity and mechanical stability in SPEs. Crosslinking establishes a three‐dimensional network that inhibits polymer crystallization and increases the amorphous phase fraction, therefore reducing the activation energy for Li‐ion transport. For instance, Sheng et al. (48) designed a crosslinked nanofiber‐reinforced PEO–PAN SPE that considerably improves lithium‐ion transport and rate capability in solid‐state lithium‐sulfur batteries. Due to PAN nanofiber‐induced crosslinking and the incorporation of polar nitrogen‐containing groups, the electrolyte demonstrates ionic conductivity of approximately 1.63 × 10^−5^ S cm^−1^ at 20°C, nearly an order of magnitude greater than that of conventional PEO‐based electrolytes, accompanied by a significantly lower activation energy. These enhancements facilitate more rapid Li‐ion diffusion and increased dissociation of lithium salts. As a consequence, Li–S cells tested at 70°C exhibit significantly enhanced rate capability, delivering approximately 1200 mAh g^−1^ at 0.1 C and maintaining over 450 mAh g^−1^ at 1 C, substantially surpassing PEO‐based equivalents.

Overall, PSEs are essential for facilitating rapid‐charging ASSLSBs, as their ionic conductivity and Li‐ion transference number directly influences their rate performance. Strategies including the integration of ceramic fillers, the optimization of filler morphology and dispersion, the design of gel or zwitterionic polymer electrolytes, and the utilization of ionic liquids effectively diminish polymer crystallinity and enhance Li‐ion transport and Li‐ion transference number, thereby resulting in improved rate performance. However, excessive filler loading, aggregation, or imbalanced solvent properties can induce transport heterogeneity and interfacial instability, thereby restricting high‐rate charge performance. Future investigations should concentrate on architected ion‐transport pathways, single‐ion or zwitterionic polymer systems that concurrently facilitate high ionic conductivity and Li‐ion transference number, thus advancing the development of durable fast‐charging ASSLSBs.

### 3.2 Mitigating Polysulfide Shuttling in Polymer‐Based SEs

Pure polymer electrolyte systems share structural similarities with LE, but they also have intrinsic disadvantages, most notably the problem of polysulfide dissolution [11]. The long‐term stability and rate retention are significantly hampered by the formation of inactive LiPS intermediate species [12]. Additionally, LiPS intermediates are partially soluble in PEO matrix, which causes dual discharge plateaus in the charge‐discharge profiles, confirms the pronounced LiPS shuttle similar to that found in ether‐based LEs [24]. Recent work has concentrated on increasing the Li‐ion transference number of PEO‐based SPEs in order to reduce LiPS shuttling. For instance, Fan et al. [33] established an effective solid/solid interfacial engineering approach employing atomic layer deposition (ALD) of ultrathin Al_2_O_3_ coatings on PEO–LiTFSI SPEs to mitigate LiPS shuttling and enhance interfacial stability in ASSLSBs. The amorphous Al_2_O_3_ layer, approximately 5 nm in thickness, served as a multifunctional barrier chemically immobilizing LiPS intermediates and physically obstructing their migration to the lithium anode. This coating impeded anion movement, elevating the Li‐ion transference number from 0.21 to 0.4–0.56, thus diminishing the concentration gradient that mitigates LiPS diffusion.

**FIGURE 4:**
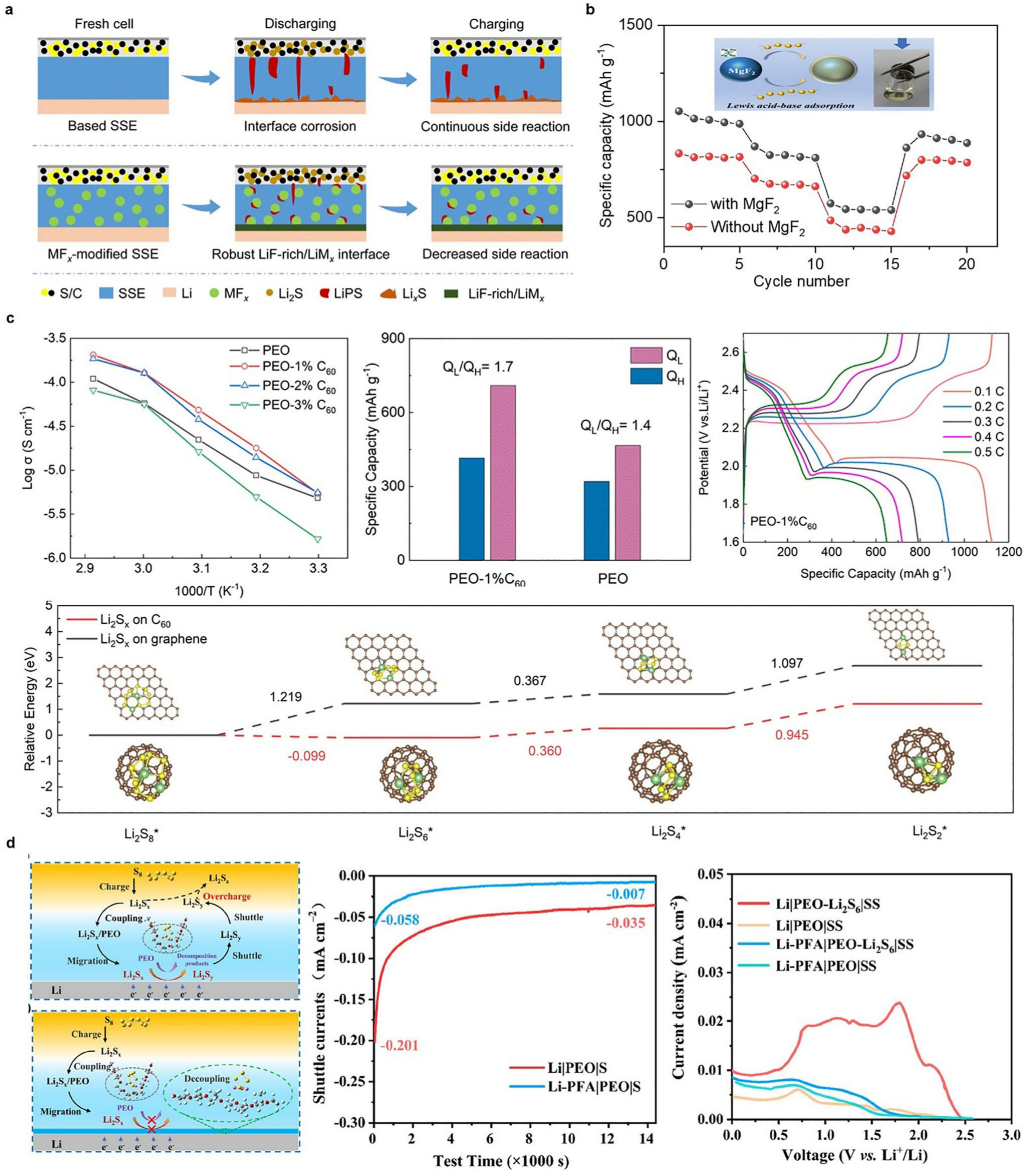
Polysulfide shuttling mitigation strategies to achieve high‐rate capability. (a,b) Effect of incorporation of MgF_2_ into PEO‐LLZTO CPE on the polysulfide shuttling and rate retention performance. Figure reproduced with permission from ref. [83] Copyright 2023, Royal Society of Chemistry. (c) Effect of addition of C_60_ to PEO on the ionic conductivity, polysulfide shuttling and rate retention behavior. Figure reproduced with permission from ref. [36] Copyright 2023, Royal Society of Chemistry. (d) Effect of incorporating F‐rich interlayer on the mitigation of polysulfide shuttle in PEO‐based SPE. Figure reproduced with permission from ref. [84] Copyright 2022, American Chemical Society.

Duan et al. [83] demonstrated that the incorporation of MgF_2_ into a PEO–LLZTO‐based SE effectively improved Li‐ion transport and electrochemical stability by simultaneously addressing LiPS shuttling. The effective confinement of LiPS species (Li_2_S_6_/Li_2_S_4_) near the cathode and the prevention of their migration toward the lithium anode were facilitated by the strong Lewis acidity of MgF_2_, which facilitated the rapid and robust chemical adsorption of these species through Mg‐S and Li‐F interactions. The coulombic efficiency was enhanced to 98.5% and side reactions were reduced as a result of the suppressed shuttle effect. Further, MgF_2_ reacted in situ with lithium to create a LiF‐rich SEI and Li_x_Mg alloy layer on the anode surface (Figure 4a). Additionally, the MgF_2_ additive decreased the crystallinity of PEO and facilitated the dissociation of LiTFSI, resulting in an increase in ionic conductivity to 4.26 × 10^−4^ S cm^−1^ at 60°C and an increase in the Li‐ion transference number to 0.187. Consequently, the modified electrolyte demonstrated enhanced Li‐ion mobility and decreased polarization during cycling. The Li‐S cells that utilized this electrolyte demonstrated exceptional rate capability across a range of current densities at 60°C (Figure 4b).

Zhang et al. [36] demonstrated that the addition of a small amount of C_60_ to a PEO‐based electrolyte effectively suppresses the polysulfide confinement in ASSLSBs. The C_60_ functioned as a redox mediator that expedited the transformation of soluble polysulfides (Li_2_S_6_/Li_2_S_4_) into insoluble Li_2_S, thereby successfully mitigating the polysulfide shuttle effect and improving electrochemical kinetics by reducing polarization and charge‐transfer resistance. In the voltage profile, the high‐order soluble polysulfides generated in the first plateau significantly contribute to the shuttle effect; thus, a larger QH for PEO–1% C_60_ signifies more effective mitigation of this phenomenon. The second plateau indicates substantial Li_2_S production, with elevated QL in conjunction with C_60_ implying more Li_2_S precipitation. The QL/QH ratio reflects polysulfide utilization efficiency, with the increased value of 1.7 for PEO–1% C_60_ (compared to 1.4 for PEO) indicating superior sulfur conversion and greater active species utilization (Figure 4c). The multifunctional effect of C_60_ in LIPS shuttling and expediting redox kinetics led to significantly enhanced ionic conductivity and rate performance.

Li et al. [38] developed a PTFE@LLZO@PEO composite SE in which a three‐dimensional porous PTFE scaffold is infiltrated with a PEO‐LLZO matrix to suppress the shuttling of polysulfides. The PTFE network establishes a dense physical barrier that effectively inhibits polysulfide migration. Cyclic voltammetry (CV) analysis reveals that the composite electrolyte produces a unified, single reduction peak, indicating more restricted sulfur redox activity and markedly reduced shuttle phenomena relative to LLZO@PEO. The design additionally enhances the stability of the Li‐metal interface, allowing for 300 h of stable symmetric cycling at a current density of 0.1 mA cm^−2^. In Li‐S cells, the electrolyte maintains 86% capacity retention after 100 cycles at 0.1 C, demonstrating excellent rate capabilities than the PEO‐LLZO matrix. These findings underscore the efficacy of scaffold‐reinforced polymer electrolytes in attaining enhanced shuttle suppression and high‐rate capabilities in ASSLSBs.

Further, the incorporation of multifunctional layers into the PSEs establishes a protective barrier that inhibits polysulfide migration. For instance, An et al. [84] established that incorporating a fluorine‐rich (F‐rich) interlayer of perfluoropolyether alcohol (PFA) between the lithium anode and the PEO‐based SPE efficiently mitigated polysulfide shuttling in ASSLSBs. The fluorine‐rich PFA layer exhibits a pronounced electron‐withdrawing action, which protects the oxygen electron cloud and spatially inhibits chemical coupling between PEO and lithium polysulfides (LiPSs). The decoupling effect impeded the infiltration of LiPSs into the electrolyte and their subsequent reduction on the lithium surface, which would otherwise result in interfacial deterioration and unstable solid electrolyte interphase (SEI) formation. Density functional theory (DFT) calculations indicated that PFA had negative solvation potentials (ΔP) with LiPSs, suggesting that polysulfides are inclined to self‐aggregate instead of dissolving or interacting with PFA, contrarily to PEO, which facilitates LiPS coupling. As a result, the F‐rich interlayer inhibited the formation of Li_2_S/Li_2_S_2_ deposits on the anode and diminished shuttle currents from −0.035 to −0.007 mA cm^−2^. Furthermore, PFA enabled the development of a LiF‐dominant solid electrolyte SEI, which offers enhanced chemical stability, consistent Li‐ion flux, and seamless Li plating and stripping. The F‐rich PFA interlayer stabilized the Li/electrolyte interface by obstructing polysulfide migration, averting PEO decomposition, and establishing a resilient LiF‐enriched solid electrolyte interphase (SEI), thereby improving both interfacial electrochemical stability and long‐term cycling durability of ASSLSBs at 60°C (Figure 4d).

Overall, recent advancements demonstrate that the incorporation of multifunctional additives and fluorine‐rich interlayers in PSEs effectively mitigates LiPS shuttling and improves the rate capabilities of ASSLSBs. However, high‐current fast‐charging operation promotes LiPS migration and interfacial polarization, rendering these strategies inadequate. Therefore, future high‐rate ASSLSBs necessitate integrated electrolyte designs that concurrently ensure effective LiPS confinement, rapid Li‐ion transport, and stable interfacial chemistry to attain enduring and high‐current operation.

### 3.3 Interface Stabilization in Polymer‐Based SEs

**FIGURE 5:**
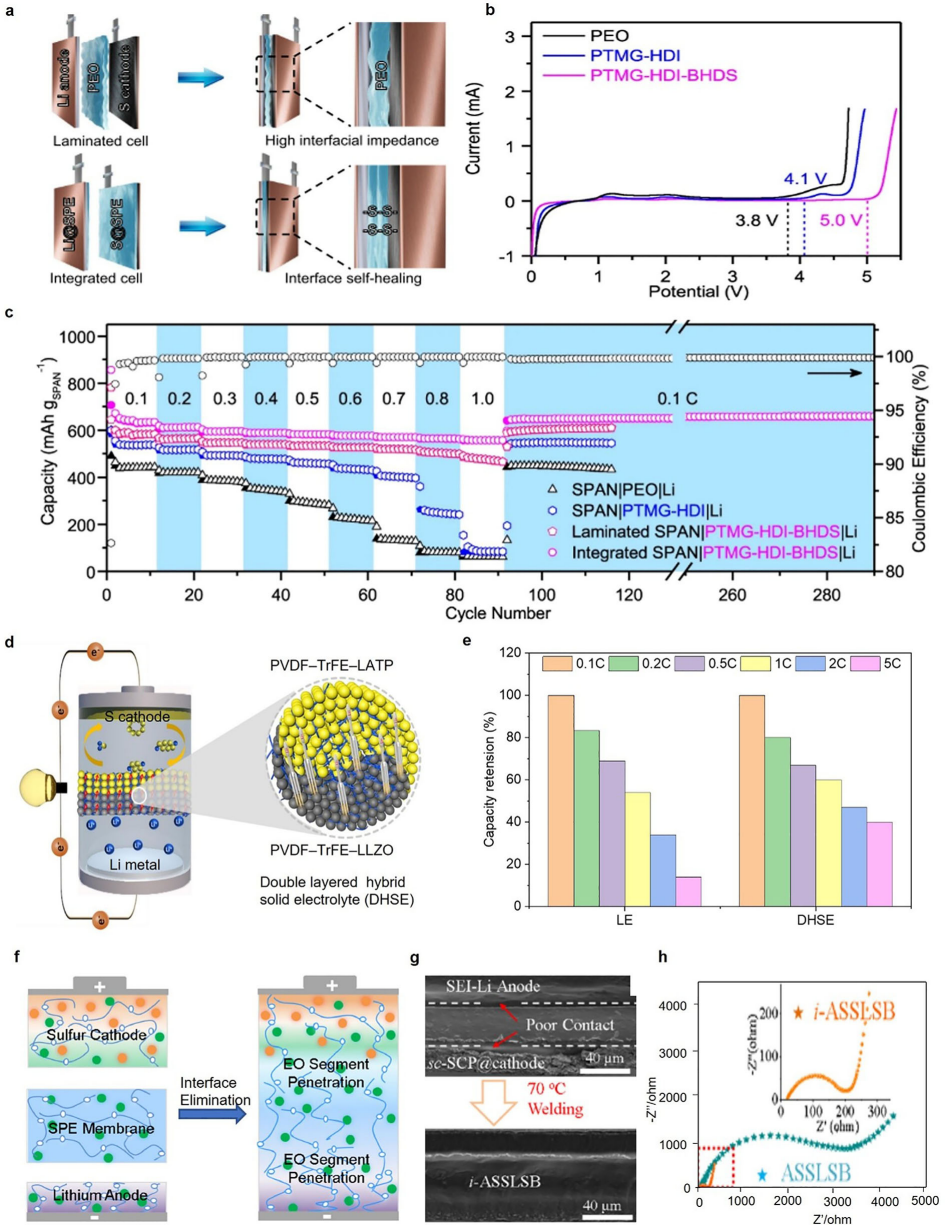
Interface engineering strategies to achieve high‐rate capability. (a–c) Effect of self‐healing polymer at the interface stabilization and rate capability retention. Figure reproduced with permission from ref. [85] Copyright 2024, Springer Nature. (d,e) Interface stabilization and rate retention behavior of double layered hybrid solid electrolyte. Figure reproduced with permission from ref. [86] Copyright 2024, Elsevier. (f–h) Interface elimination strategy and its influence on the interface resistance. Figure reproduced with permission from ref. [87] Copyright 2021, Elsevier.

Interface stability between the electrodes and PSEs is crucial for achieving high‐rate retention in ASSLSBs. A stable interface facilitates efficient Li‐ion transport, reduces charge‐transfer resistance, and preserves structural integrity at high‐current operation. Establishing robust interfacial bonding guarantees consistent ion transport, minimal polarization, and improved cycling stability crucial for the advancement of safe, high‐energy‐density Li‐S batteries. For instance, Pei et al. [85] developed a poly(ether‐urethane) SPE including dynamic disulfide and hydrogen bonding, which provided interfacial self‐healing properties, leading to remarkable electrochemical stability and rate performance in ASSLSBs. The disulfide bonds consistently reorganized to fix interfacial cracks and gaps between the electrolyte and electrodes during cycling, while the large hydrogen‐bond network preserved strong adhesion and mechanical integrity (Figure 5a). This bonding facilitated the formation of a continuous, rigid, and stable solid/solid interface that reduces interfacial resistance and prohibits dendrite development. In addition, linear sweep voltammetry (LSV) studies indicate that PTMG‐HDI‐BHDS/LiFSI possesses an extensive electrochemical stability window above 5.0 V, surpassing PEO (3.8 V) and PTMG‐HDI (4.1 V), hence proving it high electrochemical stability at the interface (Figure 5b). As a result, the full cells demonstrating an exceptional rate capability of 560 mAh g^−1^ at 1 C for the SPE containing the self‐healing properties at 30°C (Figure 5c).

Further, Liu et al. [87] developed a novel double‐layer hybrid solid electrolyte (DLHSE) to enhance the stability of the electrode‐electrolyte interfaces and improve the rate retention performance of ASSLSBs. The DLHSE comprises a NASICON‐type LATP layer in contact with the sulfur cathode and a garnet‐type LLZO layer adjacent to the lithium anode. This meticulously engineered structure improves ionic conductivity, interfacial compatibility, and mechanical strength (Figure 5d). The LATP layer exhibits elevated ionic conductivity and promotes rapid Li‐ion transport at the cathode interface, hence improving reaction kinetics and sulfur utilization. The LLZO layer establishes a chemically stable contact with lithium metal, inhibiting dendrite formation and obstructing polysulfide diffusion. The PVDF–TrFE polymer occupies interparticle spaces, creating a compact three‐dimensional network that enhances interfacial adhesion and structural stability. Simultaneously, the infiltrating ether‐based electrolyte improves ion transfer and guarantees close contact between the SE and electrodes. The DLHSE yielded reduced interfacial resistance (polarization voltage of about 16 mV) and stable lithium plating/stripping for over 500 h at 25°C and robust polysulfide confinement (Figure 5e). Further, interfacial incompatibility continues to be a significant barrier impeding the efficacy of ASSLSBs, frequently resulting in elevated resistance, sluggish ion transport, and inadequate cycling stability. Zhong et al. [87] developed an integrated cell design (i‐ASSLSB) by eliminating separate interfaces among the cathode, SPE, and anode. More interestingly, integrated cell design (i‐ASSLSB) exhibited a charge‐transfer resistance of 200 ohm, which is significantly lower than the control cell (3000 ohm) without interface modification. This design facilitates seamless Li‐ion transport, improved interfacial contact, and efficient inhibition of polysulfide shuttling by creating a continuous ion‐conducting network via polymer interpenetration, leading to higher electrochemical performance and prolonged stability at 70°C (Figure 5f–h).

The current interface‐stabilization strategies in PSEs for ASSLSBs, including self‐healing polymers with dynamic bonding, double‐layer or hybrid electrolytes, and fully integrated electrode–electrolyte architectures, effectively diminish interfacial resistance and enhance rate capability. To facilitate rapid‐charging ASSLSBs, future investigations should emphasize interface‐focused designs, such as self‐adaptive interphases, gradient or multilayer electrolytes, and integrated architectures that promote uniform Li‐ion flux, reduce polarization, and sustain stable performance under high current densities.

### 3.4 Li Dendrite Suppression in Polymer‐Based SEs

**FIGURE 6:**
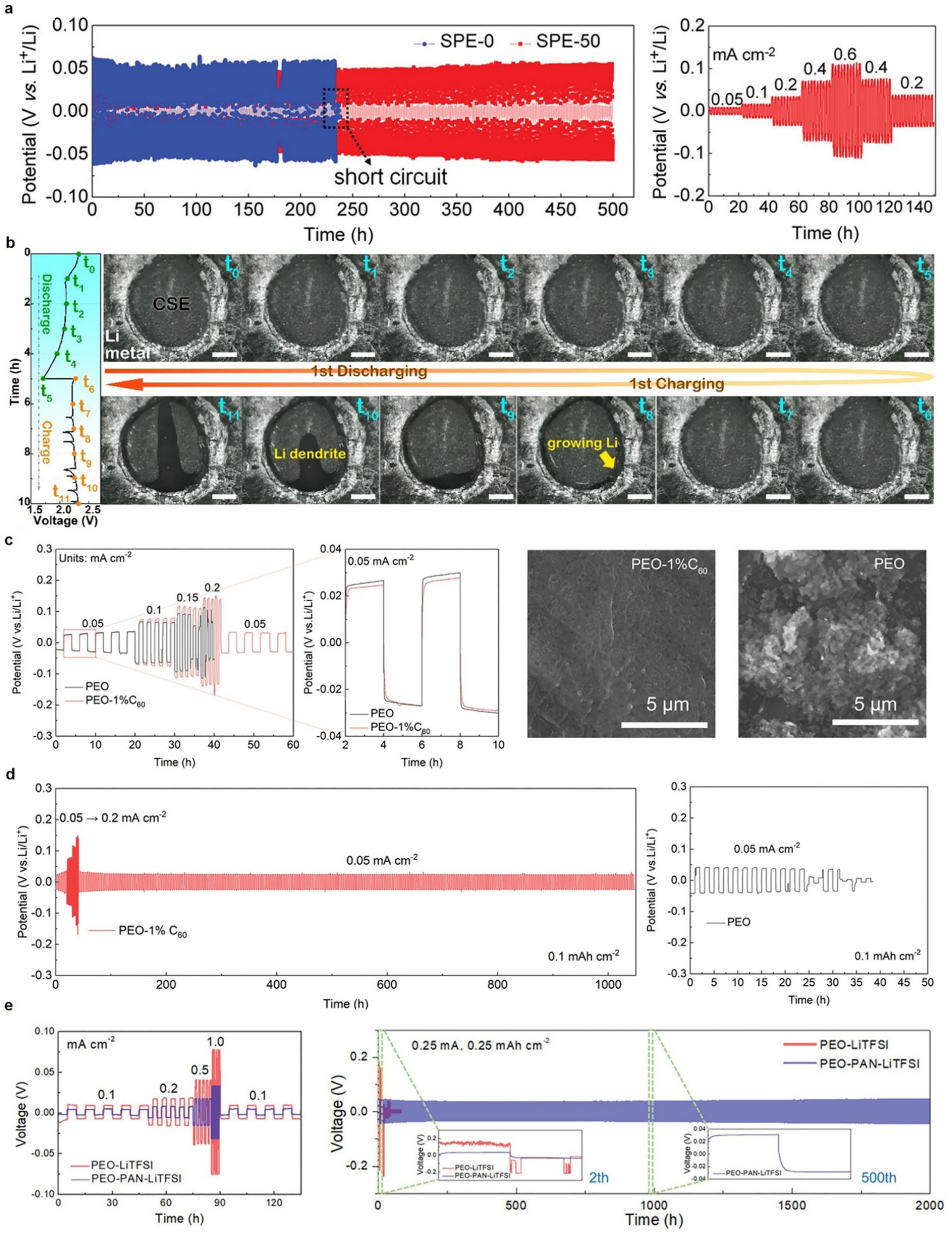
Dendrite suppression strategy in PSE‐based ASSLSBs. (a) Surface treatment strategy of PSE and its dendrite suppression Li/Li symmetric cell performance. Figure reproduced with permission from ref. [33] Copyright 2019, Wiley‐VCH. (b) Distribution of fillers in the CSE and its effective suppression of Li‐dendrite formation. Figure reproduced with permission from ref. [88] Copyright 2025, American Chemical Society. (c,d) Impact of additives in the polymer matrix on dendrite suppression. Figure reproduced with permission from ref. [36] Copyright 2023, Royal Society of Chemistry. (e) Impact of cross‐linking on the dendrite suppression performance. Figure reproduced with permission from ref. [48] Copyright 2022, Wiley‐VCH.

In PSEs, the formation of lithium dendrites is inherently associated with constraints on rapid charging, originating from ion‐transport imbalances and interfacial instability. Advanced interfacial engineering strategies such as ultrathin inorganic coatings, cross‐linking, and filler modification provide a promising approach to decouple rapid charge from dendrite formation [62, 63]. For instance, Fan et al. [33] demonstrated that the growth of lithium dendrites in PEO‐LiTFSI SPE can be effectively suppressed through the utilization of an ultrathin amorphous Al_2_O_3_ interlayer deposited via atomic layer deposition. The Al_2_O_3_ coating improves interfacial mechanical stability, elevates the Li‐ion transference number by partially impeding anion migration, and establishes a stable lithiated interphase (Li_x_Al_2_O_3_) during cycling, thereby homogenizing Li‐ion flux and facilitating uniform lithium deposition. As a result, symmetric Li/Li cells with Al_2_O_3_‐coated electrolytes demonstrate low and stable polarization (∼37 mV) and maintain dendrite‐free lithium plating and stripping for more than 500 h, whereas cells with uncoated electrolytes fail after approximately 250 h due to dendrite‐induced short circuits, thereby illustrating the efficacy of interfacial oxide engineering in suppressing lithium dendrite formation (Figure 6a). In addition, the distribution of fillers in the CSE effectively controls Li‐dendrite formation. Pan et al. [88] confirmed that the dispersion state of ceramic additives within CSEs critically influences lithium dendrite formation. Well‐dispersed LLZTO fillers establish a uniform Li‐ion conducting network, facilitating consistent interfacial Li‐ion flux and inhibiting dendritic lithium formation, whereas filler agglomeration results in spatially uneven ionic conduction and localized current‐density regions. As demonstrated by in situ optical microscopy (Figure 6b), the agglomerated‐filler electrolyte displays rapid dendrite nucleation and propagation during charge, accompanied by voltage instability, thereby establishing a direct connection between microstructural heterogeneity and dendrite‐induced failure in ASSLSBs.

Moreover, the addition of additives into the polymer network increases CCD values and suppresses the Li dendrite growth. For instance, Wei et al. [36] demonstrated that the incorporation of a small quantity of C_60_ additive (1wt%) into PEO‐based SPE markedly improves resistance to lithium dendrite formation and elevates the CCD. As depicted in Figure 6c,d, the PEO‐1% C_60_ electrolyte facilitates stable lithium plating and stripping at current densities reaching 0.2 mA cm^−2^, whereas unmodified PEO fails at 0.15 mA cm^−2^ due to dendrite‐induced short circuits. The CCD is significantly elevated from approximately 0.6 mA cm^−2^ for PEO to approximately 1.3 mA cm^−2^ with the inclusion of C_60_. This enhancement is ascribed to the elevated interfacial energy between C_60_ and lithium metal, which increases the Li nucleation barrier, facilitates lateral Li‐ion diffusion along the interface, and inhibits vertical dendrite growth into the electrolyte. Consequently, symmetric Li/Li cells with PEO‐1% C C_60_ demonstrate minimal polarization (∼22 mV) and maintain stability for over 1000 h, thereby confirming the efficacy of C_60_ as an additive for dendrite suppression. Additionally, Sheng et al. [48] demonstrated that polymer crosslinking significantly enhances the critical current density (CCD) and inhibits lithium dendrite formation by fabricating a PAN‐fiber‐reinforced, PEO‐PAN crosslinked solid electrolyte. The crosslinked network markedly improves mechanical strength while preserving high ionic conductivity and a high Li‐ion transference number, resulting in more uniform Li‐ion transport at the Li/electrolyte interface. As illustrated in Figure 6e, symmetric Li/Li cells incorporating the PEO‐PAN electrolyte demonstrate reduced polarization and maintain stable operation at elevated current densities relative to unmodified PEO electrolytes. More importantly, the crosslinked PEO‐PAN electrolyte maintains long‐term lithium plating and stripping for over 2000 h without short circuiting, whereas the non‐crosslinked PEO electrolyte deteriorates rapidly due to dendrite‐induced instability.

The inhibition of lithium dendrite in PSEs could be accomplished via the strategies, including interfacial oxide coatings, homogeneous distribution of fillers, the utilization of functional additives, and polymer crosslinking, which homogenizes Li‐ion flux, enhances mechanical strength, and increases the CCD, thereby enabling the stable lithium plating and stripping at higher current densities. Further, to achieve fast‐charging ASSLSBs, future research should focus on developing integrated electrolyte architectures that incorporate high Li‐ion transference numbers, adaptable interphases, and mechanically reinforced ion‐transport networks to ensure uniform lithium deposition and dendrite‐free operation during high‐rate charging.

## 4 Summary and Future Perspective

**FIGURE 7:**
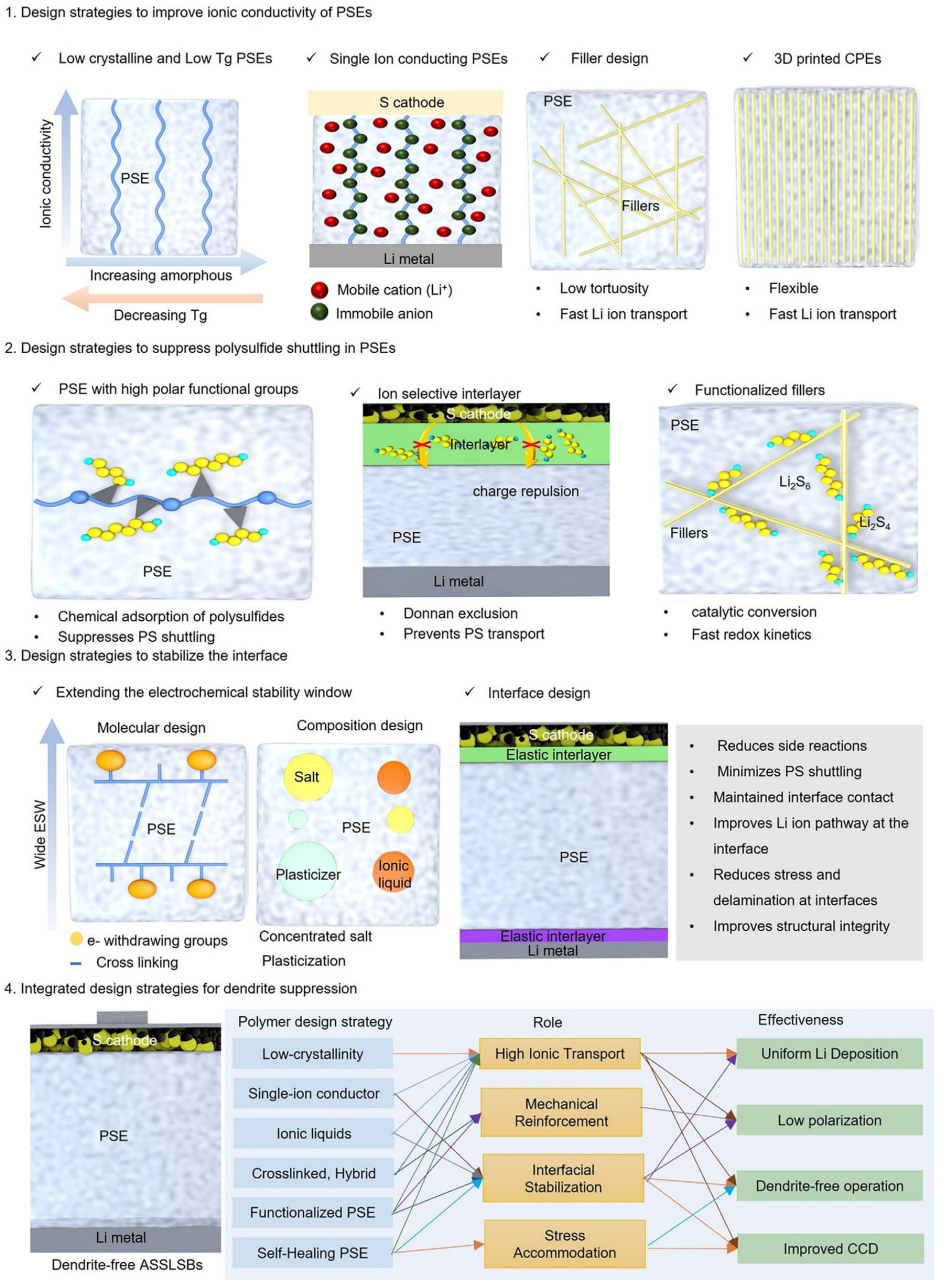
Strategy to achieve high‐rate retention and fast charging in polymer SE based ASSLSBs.

Achieving rapid charging and reliable high‐rate cycling in ASSLSBs constitutes a significant challenge in ASSLSBs. In contrast to traditional liquid systems that facilitate spontaneous ion transport and interfacial wetting, the solid‐state design is limited by slow Li‐ion mobility and significant interfacial resistance. Therefore, the rational design of PSEs must incorporate rapid ion transport, dynamic interfacial flexibility, and electrochemical stability into a unified multifunctional framework to achieve high power and fast‐charging ASSLSBs (Figure 7).

### 4.1 Design Strategies for Improving Ionic Conductivity

The achievement of high‐rate and rapid‐charging capabilities in ASSLSBs fundamentally relies on the optimization of Li‐ion transport inside the PSE matrix. A well‐engineered PSE should have elevated ionic conductivity (>10^−3^ S·cm^−1^ at 25°C) and a substantial Li‐ion transference number (>0.6) to reduce concentration polarization during fast charge–discharge cycles. Conventional PEO‐based electrolytes are constrained by their high crystallinity and modest ionic conductivity at ambient temperature. Moreover, increasing the amorphous content and lowering the glass‐transition temperature (Tg) are both effective ways to improve the ionic conductivity of PSEs. Nonetheless, lowering Tg often results in a loss of mechanical properties of the PSEs. Thus, it is critical to develop novel polymers that possess strong ionic conductivity while maintaining mechanical strength. Future research direction should focus on the utilization of copolymerization, cross‐linking, and grafting methods for designing novel PSEs electrolytes which possess reduced glass transition temperatures and enhanced Li‐ion dipole interactions, thereby facilitating improved ion dissociation and mobility under ambient conditions. In addition, advanced designs seek to separate ionic transport from polymer chain movement, facilitating more liquid‐like ion dynamics inside a solid matrix. This could be achieved by integrating ionic liquid or zwitterionic side‐chains that provide highly polar, amorphous environments functioning as fast ion‐conduction channels.

Alternatively, single‐ion conducting polymers, in which the anions are covalently bonded to the polymer backbone, efficiently mitigate anion accumulation and regulate the net Li‐ion flux at elevated current densities. Moreover, nanocomposite polymer electrolytes using ceramic fillers as LLZO or LATP nanowires have exhibited the establishment of continuous, percolated Li‐ion channels that connect polymer domains, therefore diminishing tortuosity and enhancing long‐range Li‐ion transport. The combined implementation of these strategies backbone engineering, ion‐channel creation, and inorganic–polymer coupling establishes a hierarchical transport network that can maintain elevated ionic conductivity and transference number concurrently. This transport‐centric design not only expedites charge transfer kinetics but also directly improves the limiting current density, establishing the groundwork for polymer electrolytes capable of facilitating practical fast‐charging operations in polymer‐based ASSLSBs.

Further, three‐dimensional (3D)‐printed high‐ceramic CPEs facilitate the formation of continuous ceramic networks inside a flexible polymer matrix, resolving the brittleness issues of conventional ceramic electrolytes. In addition, the percolated ceramic pathways facilitate low‐tortuosity for Li‐ion transport and enhance cation transference, significantly diminishing ion‐transport activation energy barriers and alleviating concentration polarization at high current densities. The encompassing polymer phase provides mechanical flexibility and improved interfacial contact, ensuring the integrity of CPE during cycling and facilitating high‐rate charging performance that conventional polymer electrolytes find challenging to attain.

There are numerous practical challenges in the way of developing fast‐charge PSE‐based ASSLSBs using polymer backbone engineering, ionic channel building, single‐ion conducting polymers, nanocomposite designs, and 3D‐printed ceramic frameworks. While ionic conductivity can be improved by lowering Tg and adding ionic liquids or zwitterionic side chains, these tactics frequently result in excessive polymer softening or phase separation, which impairs mechanical strength, structural stability, and long‐term durability. Although single‐ion conducting polymers increase Li‐ion transference numbers, they usually have strong Li–anion interactions that limit Li‐ion mobility and low overall ionic conductivity. Percolated ion‐transport pathways can be produced by nanocomposite electrolytes that contain ceramic fillers, however, at high ceramic loadings, these fillers cause interfacial resistance, filler agglomeration, mechanical mismatch, and processing challenges. Similar to this, continuous ion‐conduction networks are made possible by 3D‐printed high‐ceramic composite polymer electrolytes; however, these electrolytes may have brittleness, microstructural flaws, scaling issues, and higher production costs. Moreover, the fabrication of ultrathin PSE (∼50 µm) necessitates the following: precision thickness uniformity, uniform ceramic dispersion, strict defect control and sufficient densification without inducing residual stress. Additionally, scalability and reproducibility must be ensured. In addition, long‐term cycling can result in mechanical fatigue, interfacial delamination, tension accumulation, crack propagation, and degradation of the polymer network in terms of durability. These issues have the potential to disrupt ionic pathways and increase resistance over time, underscoring the necessity of addressing coupled electrochemical–mechanical stability for practical implementation. In order to provide fast‐charging PSEs for ASSLSBs, the fundamental difficulty is still to balance mechanical robustness, interfacial stability, and practical manufacturability with high ionic conductivity and Li‐ion transference number.

### 4.2 Design Strategies for Suppressing Polysulfide Shuttling

Despite significant advancements, the complete inhibition of polysulfide migration in PSEs continues to be an unresolved issue that constrains the long‐term stability and rapid charging capabilities of ASSLSBs. Future research should concentrate on molecular structure regulation, interfacial engineering, and effective functionality within the polymer matrix to suppress the polysulfide shuttling. Polymer backbones beyond conventional single‐ion conducting polymer needs to be developed in order to distinguish the selective transport between Li‐ion and polysulfide anions in the polymer matrix. In addition, the introduction of rigid ionic clusters, regulated dipole orientations, and steric confinement can create selective Li‐ion transport channels that impede polysulfide diffusion. Further, the utilization of computational screening for polymer design will expedite the identification of ion‐selective materials by forecasting solvation structures, ion‐pairing energies, and migration barriers, facilitating the targeted synthesis of polymers that integrate high Li‐ion mobility with low polysulfide solubility.

A further potential approach involves the development of self‐adaptive polymer matrices that react dynamically to electrochemical conditions. Stimuli‐responsive polymers with reversible crosslinks such as Diels–Alder bonds, hydrogen‐bonded clusters, or boronated esters may constrict or reorganize their network structure upon exposure to migratory polysulfides. This dynamic behavior enables the electrolyte to sustain elevated ionic conductivity during normal operation while autonomously limiting polysulfide diffusion under high‐current or overload scenarios. The self‐healing and self‐regulating characteristics could convert polymer electrolytes into strong barriers that actively mitigate shuttle effect impacts.

Future research should focus on designing PSE with high LiPS selective functional groups in the polymer chain. More specifically, a polymer with more Lewis‐acidic functional groups adjacent to the cathode layer could immobilize LiPS intermediates and a fluorinated or LiF‐forming anolyte layer on the anode side may serve as a selective barrier against sulfur species and stabilize the Li metal. In addition, advanced PSE fabrication method, including interfacial polymerization, layer‐by‐layer deposition, and additive manufacturing, permit meticulous regulation of composition gradients and interfacial continuity, hence reducing diffusion pathways for polysulfides. Further, in situ polymerization on the ceramic surface can establish chemically linked interfaces, minimizing voids in the PSE that often act as diffusion channels for polysulfides. In addition, an innovative strategy is the incorporating of catalytic or redox‐active species into the polymer electrolyte to facilitate the conversion of soluble polysulfides into solid Li_2_S. Integrating redox mediators, metal‐N‐C catalysts into the polymer matrix may yield a self‐regenerating electrolyte that facilitates ionic conduction and catalytic regeneration. These multifunctional electrolytes will not only mitigate the shuttle effect but also enhance redox kinetics, hence enhancing rate performance under fast‐charging conditions.

While techniques including molecular structural regulation, selective functional group incorporation, and ionic cluster design can impede LiPS diffusion, they frequently elevate Tg, diminish polymer flexibility, or obstruct Li‐ion mobility due to enhanced ion‐polymer interactions. Stimuli‐responsive and self‐adaptive PSEs are reliant on reversible chemical bonds, may experience prolonged chemical instability, bond weakness, and reduced conductivity at fast charging conditions. Introducing Lewis‐acidic groups to minimize the LiPS mobility led to an increase in interfacial resistance or excessively bind Li‐ions. Moreover, advanced fabrication techniques and in situ polymerization method needs careful control on the preparation process to avoid defects and promote scalability, the inclusion of catalytic or redox‐active species may lead to electronic leakage, side reactions, or catalyst deactivation. Consequently, attaining persistent LiPS suppression without compromising ionic conductivity, mechanical strength, interfacial stability, and industrial scalability is a significant barrier for the practical implementation of PSE in ASSLSBs.

### 4.3 Design Strategies to Improve Interface Stability

Fast‐charging ASSLSBs require electrolytes that can withstand high current densities, high Li‐ion fluxes, and transient overpotentials that readily exceed the ordinary intrinsic electrochemical stability window (ESW) of the polymer. Under these non‐equilibrium conditions, oxidative decomposition at sulfur cathodes, reductive degradation at lithium interfaces, and faster polysulfide reactions all significantly reduce cycle life. Extending the ESW must therefore enable high‐rate transport and interfacial robustness, rather than just raising bulk polymer thermodynamic breakdown thresholds. Molecular strategies include electron‐withdrawing substituents, single‐ion conducting architectures, and crosslinked or topologically restricted networks that improve oxidative and reductive tolerance while increasing transport numbers. Further, compositional modification strategies, such as using ionic‐liquid plasticization, concentrated salts, and polymer‐ceramic hybrids, could reduce parasitic reactions, reduce interfacial polarization, and enable faster Li‐ion conduction within a larger effective ESW.

In PSEs, the solid–solid interface significantly influences overall cell dynamics more than the bulk ionic conductivity. In high‐rate operation or rapid charging, the accumulation of Li‐ion and slow charge transfer at inadequately wetted interfaces cause considerable interfacial polarization, resulting in voltage hysteresis, localized current hotspots, and dendritic lithium development. Consequently, rational interface engineering must concurrently attain (1) minimal charge‐transfer resistance, (2) consistent chemical compatibility, (3) uniform Li^+^ flow distribution, and (4) mechanical accommodation of interfacial stress. The direct contact between metallic lithium and a polymer electrolyte at the lithium anode is frequently chemically unstable. Reactive anions (e.g., TFSI^−^, FSI^−^) and leftover solvent fragments may disintegrate upon contact, resulting in an inhomogeneous, resistive interphase. The essential strategy for suppression is to create a fluorine‐dense artificial solid electrolyte interphase (SEI) or interlayer that reduces interfacial energy and facilitates Li‐ion movement. These fluorine‐rich components decompose to produce LiF‐dominated SEI, characterized by high mechanical modulus, low electronic conductivity, and high Li‐ion interfacial mobility. This stabilizes lithium plating and stripping, mitigates dendrite development, and diminishes charge‐transfer resistance (R_CT_), even at ambient temperature. Thin films consisting of Li_3_N, or Li_2_O nanophases, or polymers with fluorinated segments (PVDF‐HFP, PFPE, fluorinated acrylates) exhibit notable efficacy for the interface stabilization.

Moreover, gradient‐modulus architectures featuring a rigid layer (∼1 GPa) at the lithium interface and a more pliable polymer underneath (∼100–300 MPa) can equalize lithium‐ion flux and mitigate stress during cycling. In situ polymerization on the lithium surface enhances the interfacial contact area, reducing void formation and interfacial delamination that usually result in increased overpotentials during rapid charging, thereby stabilizing the electrode structure. In addition, the sulfur cathode has issues related to sluggish redox kinetics and volumetric changes during the S ↔ Li_2_S transformation, both of them contribute to the increase in electrode resistance and impair rate capability. Interestingly, the catholyte concept resolves this issue by forming an interface‐free design that can substantially reduce the interface resistance and incorporating a thin, chemically functional polymer electrolyte layer that exhibits a great affinity for LiPS species. Polymers with cationic (e.g., quaternary ammonium, imidazolium) or polar sulfone/sulfoxide functional groups can immobilize polysulfide intermediates adjacent to the cathode, therefore reducing their migration into the bulk electrolyte.

Furthermore, the adoption of redox‐catalytic sulfur hosts like TiN, VN, or Co–N–C inside the cathode‐electrolyte interfacial zone promotes the rapid Li_2_S nucleation and dissolution, thereby alleviating kinetic polarization. These catalytic components diminish charge‐transfer barriers by offering electronically conductive, polar sites that promote interfacial redox reactions. When combined with a flexible polymer matrix that can accommodate around 80% volume change during discharge, such designed cathode‐electrolyte interfaces can maintain reversible conversion even at elevated C‐rates (>2 C), ensuring reliable capacity supply during rapid charging. Despite these advancements, attaining genuinely stable, high‐rate interfaces continues to be a frontier. Subsequent research should concentrate on chemically adaptive and spatially graded interfacial designs capable of dynamically modulating Li‐ion flux, accommodating mechanical strain, and preserving electronic insulation.

In addition, the adoption of polymer electrolytes with self‐healing properties significantly improves the interfacial stability during fast‐charge. Integrating functional precursors (e.g., fluorinated sulfonimides, nitrile, or carbonate moieties) into the polymer backbone enables controlled breakdown to produce inorganic‐rich SEI/CEI layers (LiF, Li_2_CO_3_, Li_3_N) in situ during first cycles and stabilizes the interface in the subsequent cycles even at fast‐charging current. In addition, developing PSE with functionally graded interfacial structures enhances both chemical stability and mechanical integrity. The F‐rich rigid interfacial layer at the anode can inhibit dendrite formation, but a soft, elastic inner layer guarantees intimate interfacial contact during cycling. At the cathode, a polysulfide‐attractive layer abundant in cationic sites can convert into a neutral, Li‐ion conductive bulk, establishing a dual‐phase electrolyte interface that governs the directional transport of species. This gradient structure design can be achieved through three‐dimensional printing, layer‐by‐layer polymerization, or electrophoretic deposition. Further, modifying polymer chain polarity and molecular dipoles to align with electrode work functions (lithium or sulfur) may improve charge‐transfer kinetics. This interfacial ionic methodology directly connects polymer chemistry to the electrical configuration of electrodes, facilitating low‐barrier Li‐ion transport at both surfaces.

Despite these promising interfacial and molecular design methodologies for interface stabilization, there are still several obstacles present in the practical implementation of these strategies for developing high durable fast‐charging ASSLSBs. Elevating the electrochemical stability of PSEs using strategies including, incorporation of electron‐withdrawing groups, forming crosslinked networks, or single‐ion conducting designs could reduce polymer segmental mobility and increase the interfacial resistance, limiting fast Li‐ion mobility under high current. In addition, compositional modification strategies such as ionic‐liquid plasticization or addition of concentrated will extend the effective stability window of PSEs, but they also reduce the mechanical modulus and increase prolonged degradation. Further, the incorporation of F‐rich interface or LiF‐forming systems improves interfacial stability, maintaining their uniform distribution and thickness is difficult, and excessive inorganic by‐product accumulation increases charge transfer resistance. Thus, establishing chemically stable, mechanically robust, and low‐resistance interfaces capable of sustaining high Li‐ion flux under fast‐charging conditions remains a significant practical challenge.

### 4.4 Design Strategies to Suppress Li Dendrite

Polymer design prevents lithium dendrite formation in PSEs for Li–S batteries through targeted, synergistic approaches that control ion transport, mechanical properties, and interfacial chemistry, thus facilitating fast‐charging capabilities. Ionic‐transport homogenization is accomplished through the reduction of polymer crystallinity via backbone engineering (e.g., polycarbonate‐, acrylate‐, or copolymer‐based systems) and by utilizing single‐ion conducting or zwitterionic polymers, which immobilize anions and enhance the Li‐ion transference number, thereby effectively mitigating the dendrite formation by regulating the concentration polarization and localized current density regions that induce dendrite nucleation during fast charging. In addition, mechanical strength improvement strategies, including polymer crosslinking, nanofiber reinforcement, and novel molecular architectures, enhance the elastic modulus of the electrolyte, enabling the polymer matrix to physically resist dendrite penetration while preserving interfacial flexibility. Further, interfacial stabilization is achieved through the application of functional polymer chemistries or inorganic interlayers (such as fluorinated segments, oxide coatings, or LiF‐forming additives) that facilitate the development of stable, ionically conductive, and electronically insulating interphases, thereby ensuring uniform lithium deposition. Furthermore, stress‐absorbing polymer networks, such as elastomeric or self‐healing polymers could accommodate volume changes and enhance the interfacial contact during cycling, thereby preventing fracture initiation and current concentration. Collectively, these integrated polymer design strategies separate rapid charging from dendrite development, facilitating stable, high‐rate, and durable ASSLSBs.

In addition, to achieve commercial metrics of Li‐S battery such as high sulfur loading (e.g., ≥4–6 mg cm^−^^2^, low cell resistance and ambient temperature operations for attaining high gravimetric and volumetric energy density using PSEs necessitates the integrated design strategies. The utilization of the zwitterionic gel polymer electrolytes could be a promising way because it can reduce the polysulfide migration through robust dipolar contacts, while yet preserving elevated ionic conductivity within the gel matrix, thus enabling stable battery operation at ambient temperature even under high sulfur loading conditions. In addition, the implementation of single‐ion conducting PSEs, wherein anions are fixed on the polymer backbone, resulting in an elevated Li‐ion transference number, minimizes the concentration polarization under high sulfur loading conditions. Moreover, the adaption of hybrid solid/gel polymer electrolyte structures combines the mechanical strength of SPE with the superior ionic conductivity of gel or liquid phases, enhancing ionic transport, electrode‐electrolyte interface, and overall cycling stability, while facilitating practical areal capacities. Also, the usage of thin‐film PSEs could reduce the proportion of the inactive component of the cell and allowing a higher fraction of sulfur utilization and posing minimal resistance for the Li‐ion transport, which enables more realistic battery configurations closer to commercial metrics. These methodologies collectively offer a viable approach to developing thin, high‐performance PSE systems that can accommodate high sulfur loading and stable long‐term operation, all of which are crucial for the practical commercialization of ASSLSBs.

### 4.5 Application of Theoretical and Simulation Studies

The adaption of theoretical and simulation studies is crucial in directing the rational design of PSEs for fast‐charge ASSLSBs by connecting molecular interactions to electrochemical performance. The molecular dynamics (MD) simulations enable comprehensive insights into Li‐ion transport pathways inside polymer matrices, elucidating the dependence of ion mobility on segmental chain motion, coordination environments, and polymer backbone chemistry. Analyses indicate that diminishing Li‐ion binding strength or decreasing the glass transition temperature can improve ionic conductivity, therefore alleviating polarization during high C‐rate operation. Further, density functional theory (DFT) calculations promote the quantitative investigation of interactions between functional groups of PSEs and lithium polysulfides, enabling to identify functional groups with substantial adsorption energies that efficiently mitigate polysulfide shuttling without significantly affecting the Li‐ion transport. Furthermore, phase‐field simulation studies facilitate the assessment of stress evolution and dendritic growth mechanisms at the PSE/lithium metal interface, which is especially crucial under fast charging conditions characterized by high C‐rates. Multiscale transport modeling, integrating Nernst–Planck equations with empirically obtained parameters, elucidates the collective impact of ionic conductivity, Li‐ion transference number, and tortuosity on overpotential development. Collectively, these theoretical studies provide quantitative information about diffusion coefficients, activation energies, binding energies, and stress distributions that guide the optimization of polymer chemistry, selection of functional groups, and strategies for mechanical strengthening. Thus, simulation‐guided design markedly expedites the creation of high‐conductivity, mechanically durable PSEs suitable for facilitating rapid‐charge Li–S battery systems.

## Conflicts of Interest

The authors declare no conflict of interest.

## Supporting information




**Supporting File**: advs75058‐sup‐0001‐SuppMat.docx.

## Data Availability

The authors have nothing to report.
