# Supplementary material for: Toward Fast‐Charging Polymer‐Electrolyte Based All‐Solid‐State Li–S Batteries: Insights into Limiting Factors and Perspectives
Source: Adv Sci (Weinh). 2026 Apr 9;13(27):e75058. doi: 10.1002/advs.75058 (PMC13170210; doi:10.1002/advs.75058)
Supplement: Supplementary file 1 — Supporting File: advs75058‐sup‐0001‐SuppMat.docx. [file ADVS-13-e75058-s001.docx]

**Supporting Information**

Toward Fast-Charging Polymer-Electrolyte Based All-Solid-State Li–S Batteries: Insights into Limiting Factors and Perspectives

*S. Jayasubramaniyan^1,2^, Mingxu Li^1^, Hyeok-jin Kwon^1^, Sang Yong Nam^2,3^, Younki Lee*^2,3^ Hyun Woo Kim*^1^*

^1^ Department of Chemical Engineering, Gyeongsang National University, Jinju, 52828, Republic of Korea. ^2^Research Institute for Green Energy Convergence Technology, Gyeongsang National University, Jinju, 52828, Republic of Korea

^3^Department of Materials Engineering and Convergence Technology, Gyeongsang National University, Jinju, 52828, Republic of Korea

Email: Younki Lee (ylee@gnu.ac.kr) and Hyun Woo Kim (khw5536@gnu.ac.kr)

**Table S1:** A detailed summary of material information, polymer electrolyte type, sulfur loading, and operating conditions for the cycle performance and rate test results presented in Figure 1e and 1f.

| **Cathode material** | **Type of polymer electrolyte** | **Ionic conductivity**  **of PSE (S cm**^−^**^1^)** | **Sulfur loading**  **(mg cm**^−^**^2^)** | **Initial capacity**  **(mAh g**^−^**^1^)** | **Cycling retention** | **Ref** |
| --- | --- | --- | --- | --- | --- | --- |
| S/C composite | CPE | 5.71x10^−4^ @ RT | 0.8-1 | 1211 @ 0.05C@ RT | 61% @ 100 @ 0.1C @ RT | 30 |
| S/C composite | CPE | 1.24x10^−4^ @ RT | - | 1085@ 0.2C@ RT | 71% @ 100 @ 0.2C @ RT | 31 |
| S/C composite | GPE | 7.49x10^−4^ @ 25^o^C | 1.5 | - | 88.3% @ 120 @ 0.2C @ 25^o^C | 32 |
| S/C composite | SPE | 2.5x10^−4^ @ 65^o^C | 1.2 | 1091 @ 0.05C @ 70^o^C | 62.3% @ 200 @ 0.2C @ 70^o^C | 33 |
| S/C composite | GPE | 1.19x10^−3^ @ RT | 1-1.3 | 1217 @ 0.1C @ RT | 67.5% @ 100 @0.2C@ RT | 34 |
| S@PPy | GPE | 1.87x10^−3^@ RT | 1.5 | - | 87.6% @ 150 @ 0.2C @ RT | 35 |
| S/C composite | SPE | 1.27x10^−4^ @ 60^o^C | 0.8-1 | 1148 @ 0.1C @ 55^o^C | 91.6% @ 50 @ 0.2C @ 55^o^C | 36 |
| SPAN | CPE | 0.42x10^−3^ @ 20^o^C | - | 1772 @ 0.1 @ 60^o^C | 42.5% @ 50 @ 0.1C @ 60^o^C | 37 |
| S/C composite | CPE | 2.54x10^−4^ @ 60^o^C | 1.0 | 655 @ 0.1C @ 30^o^C | 56% @ 100 @ 0.1C @ 30^o^C | 38 |
| S/C composite | SPE | 3.2x10^−4^ @ 60^o^C | - | 1131 @ 0.1 @ 60^o^C | 36.8% @ 200 @ 0.2 @ 60^o^C | 39 |
| S/C composite | CPE | 1x10^−4^ @ 50 ^o^C | 1 | 770 @ 0.1 @ 50^o^C | 84.4% @ 90 @ 0.1C @ 50^o^C | 40 |
| S/C composite | SPE | 2x10^−4^ 70^o^C | 0.8-1.0 | 1200 @ 0.05 @ 70^o^C | 65.9% @ 50 @ 0.1C @ 70^o^C | 41 |
| S/C composite | SPE | 2.62x10^−4^ @ 60^o^C | 0.5 | 1149 @ 0.5 @ 60^o^C | 40% @ 200 @ 0.5C @ 60^o^C | 42 |
| S/CNT | CPE | 5.4x10^−4^ @ 60^o^C | 0.284 | 1415 @ 0.1 @ 60^o^C | 45.2% @ 120 @ 0.1C @ 60^o^C | 43 |
| S/C composite | SPE | 1.3x10^−4^ @ 60^o^C | 0.6-0.9 | 1322 @ 0.05 @ 60^o^C | 34.2% @ 100 @ 0.1C @ 60^o^C | 44 |
| S/C composite | CPE | 2.01x10^−3^ @ 30^o^C | 1-1.5 | 1459 @ 0.1 @ 25^o^C | 58.9% @ 500 @ 1C @ 25^o^C | 45 |
| S/C composite | GPE | 1.95x10^−3^ @ 0^o^C | 1.5 | 1446 @ 0.1 @ 25^o^C | 55% @ 1000 @ 0.5C @ 25^o^C | 46 |
| S/CNT | GPE | 0.12x10^−3^ @ 25^o^C | 1.5 | 1003 @ 0.2 @ 25^o^C | 73% @ 300 @ 0.5C @ 25^o^C | 47 |
| S/BP-2000/rGO | SPE | 1.63x10^−5^ @ 20^o^C | - | 1200 @ 0.1 @ 70^o^C | 66% @ 75 @ 0.1C @ 70^o^C | 48 |
| S/C composite | SPE | >10^−4^ @ 50^o^C | 0.6 | 1034 @ 0.1 @ 55^o^C | 83% @ 100 @ 0.2C @ 55^o^C | 49 |
| SPAN | SPE | 4x10^−4^ @ RT | 1 | 1793 @ 0.1 @ 75^o^C | 80% @ 120 @ 0.1C @ 75^o^C | 50 |

CPE: composite polymer electrolyte SPE: Solid polymer electrolyte GPE: Gel polymer electrolyte

RT: Room temperature
